# Spatio-temporal data prediction of multiple air pollutants in multi-cities based on 4D digraph convolutional neural network

**DOI:** 10.1371/journal.pone.0287781

**Published:** 2023-12-22

**Authors:** Li Wang, Qianhui Tang, Xiaoyi Wang, Jiping Xu, Zhiyao Zhao, Huiyan Zhang, Jiabin Yu, Qian Sun, Yuting Bai, Xuebo Jin, Chaoran Ning

**Affiliations:** 1 Beijing Laboratory for Intelligent Environmental Protection, School of Artificial Intelligence, Beijing Technology and Business University, Beijing, China; 2 Beijing Institute of Fashion Technology, Beijing, China; TU Wien: Technische Universitat Wien, AUSTRIA

## Abstract

In response to the problem that current multi-city multi-pollutant prediction methods based on one-dimensional undirected graph neural network models cannot accurately reflect the two-dimensional spatial correlations and directedness, this study proposes a four-dimensional directed graph model that can capture the two-dimensional spatial directed information and node correlation information related to multiple factors, as well as extract temporal correlation information at different times. Firstly, A four-dimensional directed GCN model with directed information graph in two-dimensional space was established based on the geographical location of the city. Secondly, Spectral decomposition and tensor operations were then applied to the two-dimensional directed information graph to obtain the graph Fourier coefficients and graph Fourier basis. Thirdly, the graph filter of the four-dimensional directed GCN model was further improved and optimized. Finally, an LSTM network architecture was introduced to construct the four-dimensional directed GCN-LSTM model for synchronous extraction of spatio-temporal information and prediction of atmospheric pollutant concentrations. The study uses the 2020 atmospheric six-parameter data of the Taihu Lake city cluster and applies canonical correlation analysis to confirm the data’s temporal, spatial, and multi-factor correlations. Through experimentation, it is verified that the proposed 4D-DGCN-LSTM model achieves a MAE reduction of 1.12%, 4.91%, 5.62%, and 11.67% compared with the 4D-DGCN, GCN-LSTM, GCN, and LSTM models, respectively, indicating the good performance of the 4D-DGCN-LSTM model in predicting multiple types of atmospheric pollutants in various cities.

## 1. Introduction

In recent years, with the increasing level of industrialization and urbanization in China, energy consumption has risen sharply. The primary energy consumption, mainly coal, oil, and natural gas, accounts for as much as 84.7% of the total. The extensive use of fossil fuels has directly led to poor air quality and high concentrations of atmospheric pollutants in economically developed regions [1]. According to the Environmental Air Quality Standards prescribed by the Chinese government, there are six basic atmospheric pollutants, which include particulate matter and gaseous pollutants [2]: PM2.5, PM10, NO2, SO2, CO, and O3. These atmospheric pollutants have been identified as the world’s biggest environmental health risk factors by the World Health Organization. In the past decade, various provinces and cities in China have experienced extreme low visibility and severe air pollution events, and the type of atmospheric pollutants has changed from industrial smoke to finer particulate matter such as PM2.5 and PM10 [3]. Air pollution not only directly harms human health and hinders human social activities but also has negative impacts on natural ecosystems. For instance, SO2 causes soil acidification and forest degradation, PM2.5 and PM10 reduce atmospheric visibility, O3 causes significant decomposition of the global climate, and atmospheric pollutants carrying nitrogen and phosphorus elements through dry and wet deposition cause eutrophication of lakes and rivers [4]. Therefore, predicting the concentration of atmospheric pollutants is of great necessity.

Currently, there are two main types of predictive modeling methods for atmospheric pollutant concentration: mechanism-based atmospheric pollutant predictive modeling and data-driven atmospheric pollutant predictive modeling [5–7]. Mechanism-based atmospheric pollutant predictive modeling is based on atmospheric dynamics and environmental chemistry [8, 9]. It constructs mathematical models using equations to analyze the spatial and temporal distribution and diffusion of pollutants based on atmospheric pollutant emission source data and meteorological data [10, 11]. The model is then used to calculate and solve the concentration and distribution of future pollutant changes and make predictions [12–14]. For example, Liu Xiaoyong et al. constructed a Flexpart atmospheric pollution spatiotemporal diffusion model to analyze the characteristics of atmospheric pollution in the industrial cities of Beijing-Tianjin-Hebei [15]. Chen Jingfeng et al. used a Gaussian plume model to analyze the diffusion law of PM2.5 in Xi’an [16]. Yu Xiaomeng et al. used three commonly used Lagrangian atmospheric pollution diffusion models to provide further research and reference directions for environmental pollutant prediction and simulation [17]. Liu Gang et al. used nonlinear dynamics to examine the spectral distribution of atmospheric pollutant concentration time series, the fractal dimension of the time-varying curve, and the correlation dimension of the attractor in phase space for the first time [18]. However, the production and concentration of atmospheric pollutants are influenced by many factors, such as the spatial and geographical location of the pollution source and regional meteorological conditions, making it difficult to accurately establish the system structure. Therefore, relying solely on mechanism-based modeling methods is not suitable for predicting the concentration of atmospheric pollutants.

Data-driven atmospheric pollutant prediction models are built on the theories of data analysis and data mining. These models do not rely on the generation and diffusion mechanisms of pollutants; they do not depend on related physical, chemical actions, and biological processes. They are based on statistical methods and analyze the concentration changes of atmospheric pollutants and related influence factors to provide future predictions of atmospheric pollutant concentrations within a certain period [19, 20]. For example, Liu Xiaoming et al. used the VGG model to predict the concentration of PM2.5 data in the form of K-line charts, which can obtain richer information about the local variations of PM2.5 concentrations [21]. Hongbin Dai et al. proposed an Improved PCA-MEE and ISPO-LightGBM model to evaluate the hazard risk of haze in multiple cities, such as Xi’an [22]. Huang Guangqiu et al. proposed the construction of an XGBoost-MLP based on GARCH models to predict PM2.5 concentrations and fluctuations in different regions [23]. Compared to mechanism-driven methods, data-driven predictions have lower costs and offer significant advantages, especially in short-term multi-frequency predictions. However, most existing atmospheric pollutant concentration predictions are based on single data-driven models, which may not provide accurate results [11, 24, 25].

The massive, high-dimensional, spatiotemporal evolution, and multi-factor characteristics of multi-city atmospheric pollutant concentration data conform to big data features. Therefore, deep learning methods suitable for big data analysis should be applied for prediction [26]. Traditional recurrent neural networks and long short-term memory networks, when dealing with time series data, capture the short- and long-term dependencies among elements in the time series, providing guidance for the calculation of elements that are sorted later in the sequence [27]. However, the diffusion of atmospheric pollutants in multiple cities is influenced by dynamic and thermal factors, exhibiting spatial correlations [28]. Therefore, when studying urban atmospheric pollutant concentrations, it is necessary to consider the spatiotemporal correlations among surrounding cities’ atmospheric pollutant concentrations and adopt an appropriate method for modeling spatiotemporal sequences with multiple factors.

Graph convolutional neural networks (GCNs) are modeling frameworks based on graph theory that utilize deep learning for graph data, such as nodes and edges, describing pairwise relationships between nodes. Some cutting-edge models have been proposed based on GCNs, especially for urban transportation and human flow problems, providing inspiration for predicting multi-city and multi-pollutant atmospheric pollution [29, 30]. For example, Yanmin Zhu et al. proposed a GCN-DHSTNet model to measure the complicated spatial-temporal dependencies with external factors and predict crowd flow movements. In this research, cities are treated as nodes, city connections as edges, and the characterization factors of atmospheric pollution as node attributes. Traditional GCNs, when dealing with spatiotemporal sequence predictions, often only consider constructing a one-dimensional spatial distance graph to represent the spatial correlations of multi-city and multi-pollutant atmospheric pollutants. They mostly use undirected graphs to reflect the diffusion movement influences of various atmospheric pollutants among cities. Liu Juan et al. used the R-distance method to establish spatial relationships between graph nodes. Guo Kunpeng et al. used surface distances between different spatial stations to serve as edge weight information in the graph model [31]. Zhang Yuhuai et al. used three different graph structure definition methods–geographic distance, Gaussian diffusion model, and matrix training-to construct graph neural network models [32]. However, the practical inter-city relationships of atmospheric pollutants contain two-dimensional spatial relationships, including longitude and latitude, and exhibit a certain degree of directionality in pollutant diffusion. A directed graph containing two-dimensional spatial information should describe the spatial correlations among cities, while considering the multi-factor correlations and temporal dependencies of various atmospheric pollutants [33].

To address the issue of inaccurate predictions of multi-pollutant atmospheric concentrations in cities using existing techniques, this invention provides a multi-city and multi-pollutant atmospheric prediction method based on a four-dimensional directed GCN-LSTM (4D-Digraph Convolutional Network-Long and Short-Term Memory) model. This method addresses the issues of traditional GCNs in predicting multi-city atmospheric pollutant concentrations, where the graph models constructed do not consider the two-dimensional spatial relationships between cities and the directionality of atmospheric pollutant diffusion. By constructing a four-dimensional directed graph model that includes two-dimensional spatial directionality information, captures one-dimensional related information among multiple factors within graph nodes, and extracts one-dimensional related information at different temporal moments, this method provides a novel approach for predicting multi-city and multi-pollutant atmospheric concentrations with spatiotemporal characteristics.

## 2. Proposed approach

### a. Construct the graph of four-dimensional directed GCN model

Based on the geographical location of the city being studied, a four-dimensional directed GCN model graph is established. The graph is a two-dimensional directed graph, where each city is represented as a node with the node’s position being the latitude and longitude two-dimensional coordinates of the city center. Directed edges exist between nodes, represented as a planar vector pointing from one node to another.

A two-dimensional space directed graph *G* = (*ν*, *ε*) is defined, $\nu =\{v_{1}:"\mathrm{city}1",v_{2}:"\mathrm{city}2",\cdots ,v_{N}:"cityN"\}$ is the node set of the graph, and *v*_*i*_ represents the *i*th city node, *i* = 1,2,⋯,*N*. The location of the node is represented by the two-dimensional spatial information of the geographical longitude and dimension of the city center.

**Fig 1:**
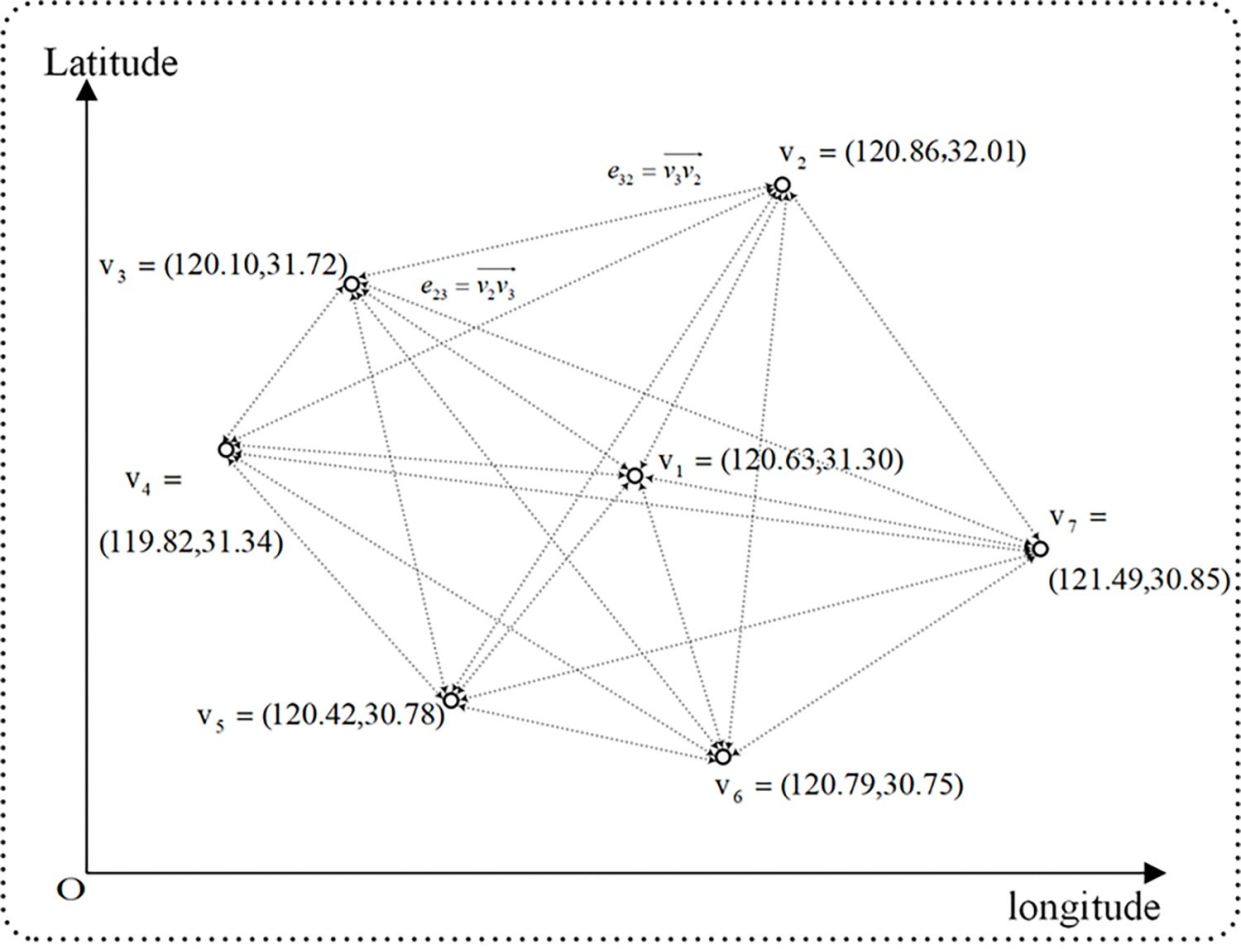
A diagram of a digraph in two dimensions.

$\varepsilon ={\{e}_{12}:\vec{v_{1}v_{2}},e_{21}:\vec{v_{2}v_{1}},\cdots ,e_{1N}:\vec{v_{1}v_{N}},e_{N1}:\vec{v_{N}v_{1}},\cdots \}$ is the set of edges between nodes, $e_{ij}:\vec{v_{i}v_{j}}$ for node *v*_*i*_ point node *v*_*j*_ plane vector, *i*,*j* = 1,2,⋯,*N*, as shown in Fig 1. Suppose the longitude and latitude coordinates of node *v*_*i*_ and *v*_*j*_ are (*x*_*vi*_, *y*_*vi*_) and (*x*_*vj*_, *y*_*vj*_) respectively, then $e_{ij}:\vec{v_{i}v_{j}}=(x_{vj}-x_{vi},y_{vj}-y_{vi})$. There are directed edges between all nodes.

Each atmospheric pollutant is regarded as a node attribute with a value that changes over time. A two-dimensional directed graph is constructed, and its adjacency matrix, degree matrix, and Laplacian matrix are established. The adjacency matrix describes the information of nodes and edges between them, while the degree matrix represents the sum of weights between nodes and their connected nodes. The time series concentration data of various atmospheric pollutants across multiple cities can be represented as a sequence of information of the two-dimensional directed graph.

Given A two-dimensional spatial directed graph *G* = (*ν*, *ε*), the adjacency matrix ***A*** is defined to describe the information of nodes and edges between nodes in the graph. The element ***A*** in row *i* and column *j* of the adjacency matrix ***A*** represents the connection relation between nodes *v*_*i*_ and *v*_*j*_. Consideration must be given to the impact of pollutant diffusion from neighboring cities on the concentration of atmospheric pollutants in each city. In other words, the spatial relationships between different city nodes influence the concentration of atmospheric pollutants in the city. To model the edge information between nodes, an adjacency matrix is constructed using the latitude and longitude coordinates of each city, represented as a planar vector. Each city node possesses a unique latitude and longitude coordinate, and the edge relationship between any two cities is represented as a planar vector constructed from the difference between the respective latitude and longitude coordinates. The specific adjacency matrix, denoted as matrix ***A***, is as follows:

$$A=\left(\begin{array}{ccc} A_{11} & \cdots & A_{1N}\\ \vdots & \ddots & \vdots \\ A_{N1} & \cdots & A_{NN} \end{array}\right)=\left(\begin{array}{ccc} 0 & \cdots & \underset{v_{1}v_{N}}{\to }\\ \vdots & \ddots & \vdots \\ \underset{v_{N}v_{1}}{\to } & \cdots & 0 \end{array}\right)$$

$$=\left(\begin{array}{ccc} \left(0,0\right) & \cdots & \left(x_{vN}-x_{v1},y_{vN}-y_{v1}\right)\\ \vdots & \ddots & \vdots \\ \left(x_{v1}-x_{vN},y_{v1}-y_{vN}\right) & \cdots & \left(0,0\right) \end{array}\right)$$

Where *v*_*i*_ and *v*_*j*_ represent the latitude and longitude coordinates of city *i*.

The degree matrix ***D*** is defined to describe the sum of weights between nodes and adjacent nodes in the graph *G* = (*ν*, *ε*), which corresponds to the adjacency matrix one by one. The degree matrix ***D*** is a diagonal matrix, that is, except the element ***D***_*ii*_ in row *i* and column *i* represents the sum of weights of edges related to node *i*, the other positional elements are 0, *i* = 1,2,⋯,*N*. The degree matrix ***D*** is as follows:

$$D=\left(\begin{array}{ccc} D_{11} & \cdots & 0\\ \vdots & \ddots & \vdots \\ 0 & \cdots & D_{NN} \end{array}\right)=\left(\begin{array}{ccc} \sum_{i=2}^{N}\left|A_{1i}\right) & \cdots & 0\\ \vdots & \ddots & \vdots \\ 0 & \cdots & \sum_{i=1}^{N-1}\left|A_{Ni}\right| \end{array}\right)$$ (1)

Then:

$$\sum_{i=2}^{N}\left|A_{1i}\right|=(\left|x_{v2}-x_{v1}|+\cdots +|x_{vN}-x_{v1}|,|y_{v2}-y_{v1}|+\cdots +|y_{vN}-y_{v1}\right|)$$

$$\sum_{i=1}^{N-1}\left|A_{Ni}\right|=(\left|x_{v1}-x_{vN}|+\cdots +|x_{v(N-1)}-x_{vN}|,|y_{vN}-y_{v1}|+\cdots +|y_{vN}-y_{v(N-1)}\right|)$$

Define the Laplacian matrix ***L*** as the difference between the degree matrix ***D*** and the adjacency matrix ***A***. Laplace matrix ***L*** is calculated by the following formula:

$$L=D-A=\left(\begin{array}{ccc} \sum_{i=2}^{N}\left|A_{1i}\right) & \cdots & \left(-(x_{vN}-x_{v1}),{-(y}_{vN}-y_{v1})\right)\\ \vdots & \ddots & \vdots \\ \left(-(x_{v1}-x_{vN}),-(y_{v1}-y_{vN})\right) & \cdots & \sum_{i=1}^{N-1}\left|A_{Ni}\right) \end{array}\right)$$ (2)

**Fig 2:**
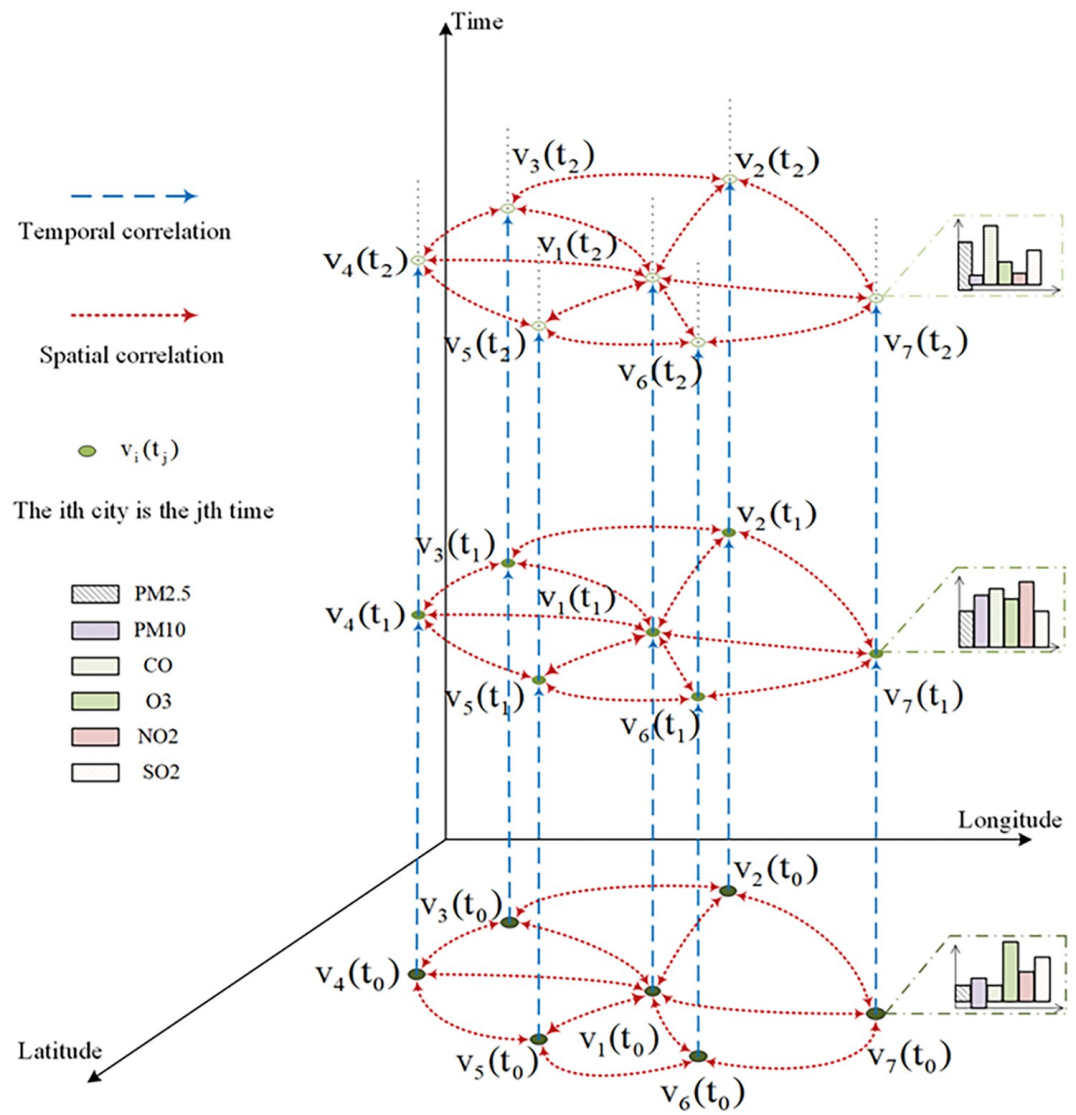
Diagram of a four-dimensional digraph.

The concept of the two-dimensional directed graph serves as the key to understanding the graph of the four-dimensional directed GCN model since it contains the spatial relation graph, which is a two-dimensional directed graph. The spatio-temporal series of different air contaminants in several cities are created into graph data form, as shown in Fig 2.

### b. Spectral decomposition and tensor operation of four-dimensional directed GCN model

In the traditional frequency domain graph convolutional neural network, the condition of frequency domain convolution for the Laplacian matrix ***L*** of the graph is that the graph is undirected, that is, the graph’s degree matrix ***D*** and adjacency matrix ***A*** are symmetric matrices.

The spectral decomposition of the four-dimensional directed GCN model is to construct a new adjacency matrix ***A***′ from the adjacency matrix ***A*** of the two-dimensional space directed graph by linear algebraic transformation. The new adjacency matrix ***A***′ is a symmetric matrix, containing the information of the old degree matrix ***D*** and the old adjacency matrix ***A***. According to the new adjacency matrix ***A***′ renewal matrix ***D***′, a new symmetric Laplace matrix ***L***′ is obtained. Spectral decomposition of the new Laplace matrix ***L***′ is carried out to obtain the eigenvalue matrix **Λ** and eigenvector matrix ***U***, which are used as the graph Fourier coefficients and graph Fourier bases respectively, and can carry out graph convolution operation in the spectral domain.

According to the property of antisymmetric matrix, if ***D*** is A symmetric matrix and ***A*** is an antisymmetric matrix, then the following relation exists:

$$D=D^{T},A={-A}^{T}$$

$$(DA-AD)={\left(DA-AD\right)}^{T}$$ (3)

The top script T represents the transpose of the matrix.

The new adjacency matrix ***A***′ = ***DA***−***AD*** composed of ***A*** and ***D*** is a symmetric matrix, then the update degree matrix ***D***′ is as follows:

$$D^{\prime }=\left(\begin{array}{ccc} D_{11}^{\prime } & \cdots & 0\\ \vdots & \ddots & \vdots \\ 0 & \cdots & D_{NN}^{\prime } \end{array}\right)=\left(\begin{array}{ccc} \sum_{j=1}^{N}A_{1j}^{\prime } & \cdots & 0\\ \vdots & \ddots & \vdots \\ 0 & \cdots & \sum_{j=1}^{N}A_{Nj}^{\prime } \end{array}\right)$$

Update the Laplacian matrix ***L***′ as follows:

$$L^{\prime }=D^{\prime }-A^{\prime }=D^{\prime }-(DA-AD)$$ (4)

The updated Laplace matrix ***L***′ is a symmetric matrix and can be decomposed spectral. After spectral decomposition, eigenvalue matrix **Λ** and eigenvector matrix ***U*** are obtained as follows:

$$\Lambda =\left(\begin{array}{ccc} {(\lambda }_{x_{1}},\lambda_{y_{1}}) & \cdots & 0\\ \vdots & \ddots & \vdots \\ 0 & \cdots & {(\lambda }_{x_{N}},\lambda_{y_{N}}) \end{array}\right)$$

$$U=(\mu_{1},\mu_{2},\cdots ,\mu_{N})$$

$$=\left(\begin{array}{ccc} \left(\mu_{x_{11}},\mu_{y_{11}}\right) & \cdots & \left(\mu_{x_{1N}},\mu_{y_{1N}}\right)\\ \vdots & \ddots & \vdots \\ {(\mu_{x_{N1}},\mu }_{y_{N1}}) & \cdots & \left(\mu_{x_{NN}},\mu_{y_{NN}}\right) \end{array}\right)$$

Where, $\left(\lambda_{x_{i}},\lambda_{y_{i}}\right)$ represents the *i*th eigenvalue of matrix ***L***′, ***μ***_*i*_ represents the eigenvector corresponding to the *i*th eigenvalue, $\left(\mu_{x_{ij}},\mu_{y_{ij}}\right)$ represents the *i*th element in the *j*th eigenvector.

The input data of the traditional GCN model is a two-dimensional matrix ***H***, as follows:

$$H=\left(\begin{array}{ccc} h_{11} & \cdots & h_{1M}\\ \vdots & \ddots & \vdots \\ h_{N1} & \cdots & h_{NM} \end{array}\right)$$

Where, matrix ***H*** is a two-dimensional matrix of N*M, and *h*_*ij*_ represents the input data of the *j*th air pollutant in the *i*th city to the traditional GCN model, *i* = 1,2,⋯,*N*, *j* = 1,2,⋯,*M*.

For the four-dimensional directed GCN model, the matrices **Λ** and ***U*** are N*N*2 three-dimensional matrices, and the problem of tensor dimension mismatch exists when the matrix **Λ** and ***U*** perform tensor operation with matrix ***H***. Therefore, the Numerical Python library broadcasting mechanism is used to extend the dimension of ***H*** and copy elements, and ***H*** is transformed into the input data of the four-dimensional directed GCN model, as follows:

$$H=\left(\begin{array}{ccc} {{(h}_{11},h_{11})}^{T} & \cdots & {{(h}_{1M},h_{1M})}^{T}\\ \vdots & \ddots & \vdots \\ {{(h}_{N1},h_{N1})}^{T} & \cdots & {{(h}_{NM},h_{NM})}^{T} \end{array}\right)$$ (5)

The transformed matrix ***H*** is a three-dimensional matrix of N*M*2, and (*h*_*NM*_, *h*_*NM*_)^T^ represents the broadcasting stretching and transposing of elements *h*_*NM*_ in the third dimension.

Transform into a three-dimensional matrix with the same dimension as the eigenvector matrix, and perform tensor operation between the transformed input matrix and the eigenvector matrix to integrate the directional information of two-dimensional space; The transformed ***H*** is a three-dimensional matrix, and the formula for tensor operation of dot product with ***U*** is as follows:

$$U∙H=(\begin{array}{ccc} \left(\mu_{x_{11}},\mu_{y_{11}}\right) & \cdots & \left(\mu_{x_{1N}},\mu_{y_{1N}}\right)\\ \vdots & \ddots & \vdots \\ {(\mu_{x_{N1}},\mu }_{y_{N1}}) & \cdots & \left(\mu_{x_{NN}},\mu_{y_{NN}}\right) \end{array})∙(\begin{array}{ccc} {{(h}_{11},h_{11})}^{Τ} & \cdots & {{(h}_{1M},h_{1M})}^{Τ}\\ \vdots & \ddots & \vdots \\ {{(h}_{N1},h_{N1})}^{Τ} & \cdots & {{(h}_{NM},h_{NM})}^{Τ} \end{array})$$ (6)

The input matrix is a matrix obtained according to the concentration data of multiple air pollutants in multiple cities.

### c. Improved graph filter for four-dimensional directed GCN model

The graph Fourier basis and the graph Fourier coefficient are discovered after the Fourier transform of the graph signal and its conversion to the frequency domain. By modulating the graph filter, the graph Fourier coefficient is altered. Traditional graph filters use the Chebyshev filter, however there will be significant variations near the passband frequency, leading to unstable signal processing. The issue of noise interference in the end portion of the signal is therefore resolved by the present invention, which employs the Hermite filter as a graph filter to filter the converted input matrix.

Define the graph filter polynomial as ***g***_***θ***_(***Λ***), as follows:

$$g_{\theta }(\Lambda )=\sum_{\kappa =0}^{K}\theta_{\kappa }\Lambda^{\kappa }=\theta_{0}\Lambda^{0}+\theta_{1}\Lambda^{1}+\cdots +\theta_{K}\Lambda^{K}$$ (7)

Where ***θ***_***κ***_ represents the coefficient of the filter, ***K*** represents the graph filter polynomial truncation parameter, used to modulate the graph input data ***H***, Feature transformation is realized by convolution operation between data ***H*** and graph filter ***g***_***θ***_(***Λ***). The specific formula is as follows:

$$H*g_{\theta }(\Lambda )$$

$$=Ug_{\theta }(\Lambda )U^{T}∙H$$

$$=U(\sum_{\kappa =0}^{K}\theta_{\kappa }\Lambda^{\kappa })U^{T}∙H$$

$$=\sum_{\kappa =0}^{K}\theta_{\kappa }{U\Lambda }^{\kappa }U^{T}∙H$$ (8)

However, the bases {**Λ**^0^, **Λ**^1^, **Λ**^2^,⋯,**Λ**^*K*^ } of the graph filter polynomial *g*_*θ*_(**Λ**) are not orthogonal to each other, that is, the coefficients are interdependent, and each part of the basis is easy to interfere with each other during model training. Therefore, Hermite polynomial with orthogonality is used to update the graph filter, and the recursive relation of Hermite polynomial is as follows:

$${Her}_{k}(\Lambda )=2\Lambda ∙{Her}_{k-1}(\Lambda )-2(k-1){Her}_{k-2}(\Lambda )$$ (9)

Where, *k*≥2, *Her*_0_(**Λ**) = **Λ**^0^, *Her*_1_(**Λ**) = 2**Λ**

Substituting Eq (9) into Eq (7), we get:

$$g_{\theta }^{\prime }(\Lambda )=\sum_{\kappa =0}^{K}\theta_{\kappa }{Her}_{k}(\Lambda )$$

$$=\theta_{0}{Her}_{0}(\Lambda )+\theta_{1}{Her}_{1}(\Lambda )+\cdots +\theta_{K}{Her}_{K}(\Lambda )$$ (10)

Substitute Eq (10) into Eq (8) and use Hermite polynomial filter for matrix ***H***. The specific calculation is shown in Eq (11):

$$ST=H*g_{\theta }^{\prime }(\Lambda )=Ug_{\theta }^{\prime }(\Lambda )U^{T}∙H$$

$$=U(\sum_{\kappa =0}^{K}\theta_{\kappa }{Her}_{k}(\Lambda ))U^{T}∙H$$

$$=\sum_{\kappa =0}^{K}\theta_{\kappa }{U{Her}_{k}(\Lambda )U}^{T}∙H$$ (11)

Where, ***ST*** represents the output data of Hermite polynomial filter.

### d. The four—Dimensional directed GCN-LSTM model is constructed

The concentration data of various atmospheric pollutants across multiple cities not only exhibit spatial correlation, i.e., a two-dimensional directed structure, but also possess temporal and multi-factorial correlation. The four-dimensional directed GCN model with its two-dimensional directed graph structure can reveal the spatial correlation between multi-factorial data. However, it is equally crucial to extract information regarding the temporal and multi-factorial correlation among the data.

**Fig 3:**
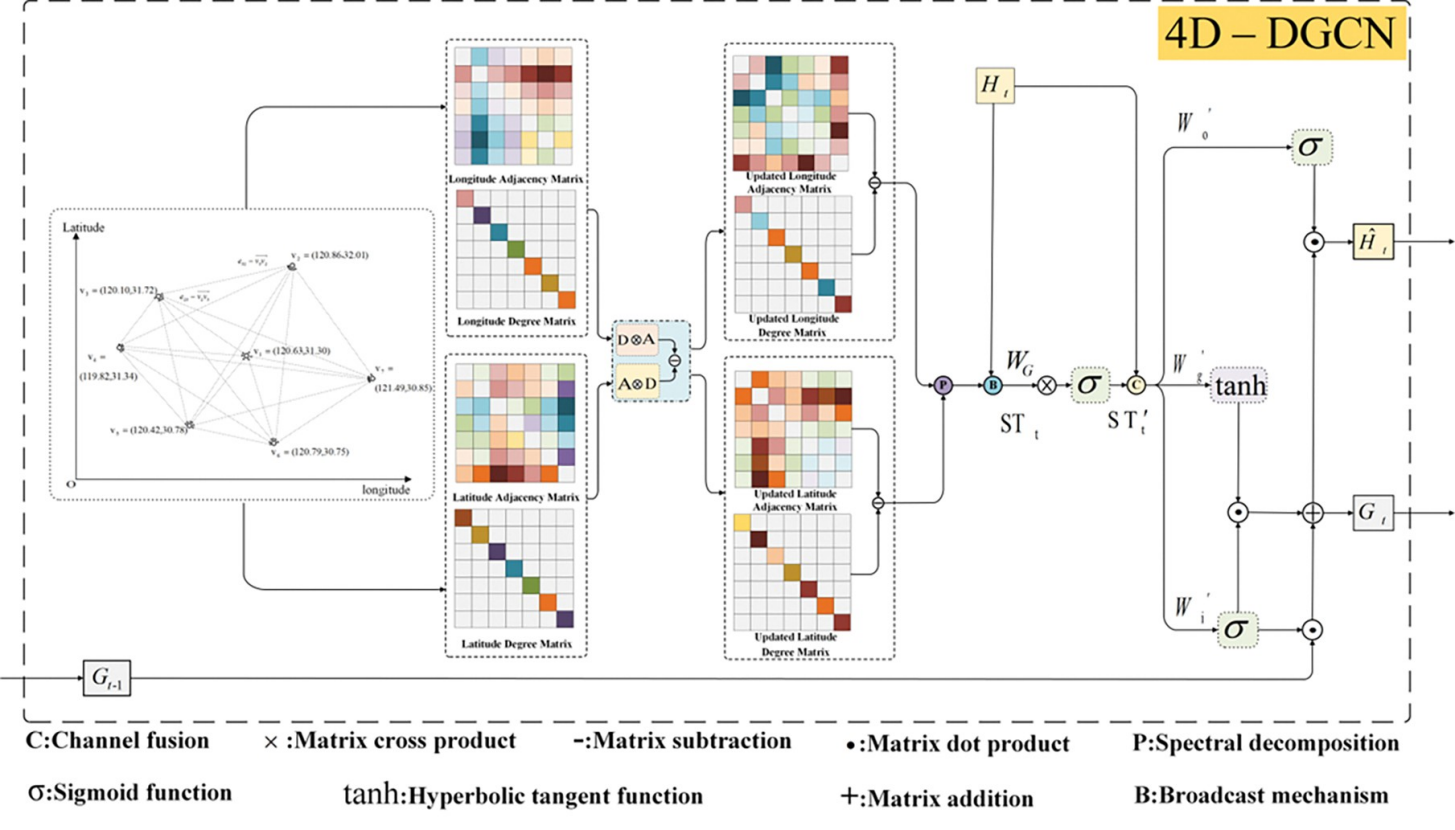
Structure diagram of four-dimensional directed GCN model.

Firstly, the calculation formula of the four-dimensional directed GCN model is explained. The concentration matrix of multiple air pollutants in multi-cities outputs the input matrix of the four-dimensional directed GCN model through the LSTM model. The filtered data can be obtained by the input matrix ***H***_***t***_ through step three-dimensional transformation and step four filtering at each time point. Set the input data at time t after filtering as ***ST***_***t***_. The output data ***ST***_***t***_ of Hermite polynomial filter was passed into the four-dimensional directed GCN model network layer for weight and bias training. The network structure of the four-dimensional directed GCN model is shown in Fig 3.

As shown in Fig 3, the structure and implementation functions of the four-dimensional directed GCN model mainly include: Information is extracted from the longitude and dimension of the two-dimensional directed graph of the four-dimensional GCN model to obtain the adjacency matrix, degree matrix and Laplace matrix. The spectral decomposition of the Laplace matrix is carried out to obtain the eigenvalue matrix and eigenvector matrix, corresponding to the graph Fourier coefficient and the graph Fourier basis respectively. The graph filter modulates the graph Fourier coefficient. The input data ***H***_***t***_ is filtered, and the filtered data ***ST***_***t***_ is output ${ST}_{t}^{\prime }$ through the network layer training of the four-dimensional directed GCN model, and sent into the input gate, oblivion gate and output gate together with the input data ***H***_***t***_. Combined with the output $i_{t}^{\prime }$ of the input gate and the output $g_{t}^{\prime }$ of the forgetting gate, the integrated output ***G***_***t***_ of the short-term memory and long-term memory of the spatial characteristics at time ***t*** is obtained. From the output $O_{t}^{\prime }$ and ***G***_***t***_ of the output gate, the output data ${\hat{H}}_{t}$ of the four-dimensional directed GCN model at time t is obtained.

The calculation formula of the four-dimensional directed GCN model is as follows:

$${ST}_{t}=\sum_{\kappa =0}^{K}\theta_{\kappa }{U{Her}_{k}(\Lambda )U}^{T}∙H_{t}$$ (12)

$${ST}_{t}^{\prime }=Sigmoid({ST}_{t}*W_{G}+b_{G})$$ (13)

$$i_{t}^{\prime }=Sigmoid(W_{i}^{\prime }*[{ST}_{t}^{\prime },H_{t}]+b_{i}^{\prime })$$ (14)

$$i_{t}^{\prime }=Sigmoid(W_{i}^{\prime }*[{ST}_{t}^{\prime },H_{t}]+b_{i}^{\prime })$$ (15)

$$G_{t}={(1+i}_{t}^{\prime })∙G_{t-1}+i_{t}^{\prime }∙g_{t}^{\prime }$$ (16)

$$O_{t}^{\prime }=Sigmoid(W_{o}^{\prime }∙[{ST}_{t}^{\prime },H_{t}]+b_{o}^{\prime })$$ (17)

$${\hat{H}}_{t}=O_{t}^{\prime }+G_{t}$$ (18)

***H***_***t***_ represents the input data of the four-dimensional directed GCN model at time ***t*, *ST***_***t***_ represents the output data of Hermite polynomial filter at time ***t*, *W***_***G***_***、b***_***G***_ represent the weight and bias of convolution operations in the four-dimensional directed GCN model, respectively. ${ST}_{t}^{\prime }$ represents the four-dimensional directed GCN model network layer training output at time $t,W_{i}^{\prime }、b_{i}^{\prime }$ represent the weight and bias of input gates in the 4D directed GCN model, respectively. $i_{t}^{\prime }$ represents the output of the input gate in the four-dimensional directed GCN model at time $t,W_{g}^{\prime }、b_{g}^{\prime }$ represent the weight and bias of forgetting gate in 4D directed GCN model, respectively. $g_{t}^{\prime }$ represents the output of the forgetting gate in the four-dimensional directed GCN model at time $t,W_{o}^{\prime }、b_{o}^{\prime }$ represent the weight and bias of the output gate in the 4D directed GCN model, respectively. $O_{t}^{\prime }$ represents the output of the output gate in the four-dimensional directed GCN model at time ***t*, *G***_***t***−**1**_ represents the output of the integration of long short-term memory of the spatial features at time ***t***−**1**, ***G***_***t***_ represents the integrated output of short-term memory and long-term memory of spatial features at time ***t***, and ${\hat{H}}_{t}$ represents the output data of the four-dimensional directed GCN model at time ***t***.

**Fig 4:**
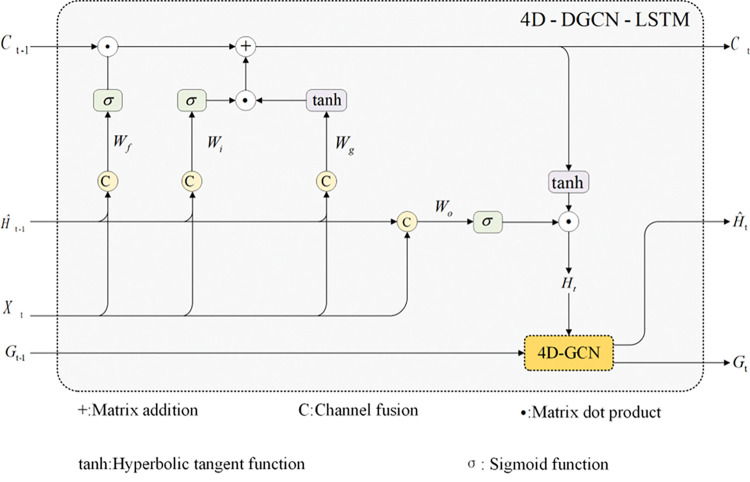
Structure diagram of four-dimensional directed GCN-LSTM model.

Then, the long short-term memory cycle structure of the LSTM is used to extract the time sequence features of various factors at various points after the four-dimensional directed GCN model has been integrated into the LSTM network architecture. In order to describe both the spatial and temporal correlation of the multi-factor data simultaneously, the spatial aspects of the graph data are extracted while the time sequence relations of the multi-factor data are preserved. Fig 4 illustrates the precise organization of the four-dimensional directed GCN-LSTM model that simultaneously collects multi-factor temporal and spatial information.

As shown in Fig 4, time series of concentrations of various air pollutants in multiple cities are obtained. Input the concentration data ***X***_***t***_ into the LSTM model together with the output data ${\hat{H}}_{t-1}$ of the four-dimensional directed GCN model at the same time. The LSTM model then outputs ***H***_***t***_ onto the 4D directed GCN model.

The formula for calculating the LSTM structure graph in the four-dimensional directed GCN-LSTM model is as follows:

$$f_{t}=Sigmoid(W_{f}∙[X_{t},{\hat{H}}_{t-1}]+b_{f})$$ (19)

$$i_{t}=Sigmoid(W_{i}∙[X_{t},{\hat{H}}_{t-1}]+b_{i})$$ (20)

$$g_{t}=tanh(W_{g}∙[X_{t},{\hat{H}}_{t-1}]+b_{g})$$ (21)

$$O_{t}=Sigmoid(W_{o}∙[X_{t},{\hat{H}}_{t-1}]+b_{o})$$ (22)

$$C_{t}=f_{t}∙C_{t-1}+i_{t}∙g_{t}$$ (23)

$$H_{t}=O_{t}∙tanh(C_{t})$$ (24)

Where ${\hat{H}}_{t-1}$ respectively represents the output of the four-dimensional directed GCN-LSTM model at time *t*−1, ***X***_*t*_ represents the concentration matrix of atmospheric pollutants at time *t*, ***f***_*t*_ represents the output of forgetting gate in LSTM network at time *t*, ***W***_*f*_、***b***_*f*_ represent the weight and bias of forgetting gate in LSTM network respectively. ***i***_*t*_ represents input gate output in LSTM network at time *t*, ***W***_*i*_和***b***_*i*_ represent weight and bias of input gate and forgetting gate respectively in LSTM network. ***g***_*t*_ represents an intermediate write information, ***W***_*g*_和***b***_*g*_ represent the weight and bias of the intermediate write information network respectively, ***O***_*t*_ represents output gate output in LSTM network at time *t*, ***W***_*o*_、***b***_*o*_ represent weight and bias of output gate in LSTM network respectively, ***C***_*t*−1_ and ***C***_*t*_ represent the *t*−1 time state and *t* time state of a memory unit in LSTM network, respectively. *Sigmoid* and *tanh* are two activation functions used in model network training, respectively.

## 3. Experimental data and parameter optimization

### a. Datasets

Taking the data of six major air pollutants, PM2.5, PM10, NO2, SO2, CO, and O3, in the Yangtze River Delta and Beijing-Tianjin-Hebei urban agglomerations as examples, this study predicts these six pollutants. The Yangtze River Delta urban agglomeration includes Suzhou, Nantong, Changzhou, Yixing, Huzhou, Jiaxing, and Shanghai, while the Beijing-Tianjin-Hebei urban agglomeration includes Beijing, Tianjin, Baoding, Datong, Zhangjiakou, Chengde, and Qinhuangdao. Using the 2020 urban meteorological monitoring data as an example, after data screening, a total of seven cities’ data were selected, which includes six atmospheric parameters. The time span of the data is from January 1, 2020, to December 31, 2020, a total of 366 days, with data collected every three hours, making it 8 time points per day. The data dimensions are (2928, 7, 6), which represents the length of time points is 2928, there are 7 cities, and 6 atmospheric pollutants. The specific information of the obtained data set is shown in Tables 1 and 2.

**Table 1:** Yangtze River Delta urban agglomeration data set.

Attribute	Number	Content
Number of nodes	Seven	Suzhou, Nantong, Changzhou, Yixing, Huzhou, Jiaxing, Shanghai
Feature of nodes	Six	PM2.5、PM10、CO、O_3_、NO_2_、SO_2_
Length of Time	In 2020	366 days×8 PCS/day = 2928
Division of Time	(1,330)、(331,360)	Day 1–330 training set, Days 331–360 test set

**Table 2:** Beijing-Tianjin-Hebei urban agglomeration data set.

Attribute	Number	Content
Number of nodes	Seven	Beijing, Tianjin, Baoding, Datong, Zhangjiakou, Chengde, Qinhuangdao
Feature of nodes	Six	PM2.5、PM10、CO、O_3_、NO_2_、SO_2_
Length of Time	In 2020	366 days×8 PCS/day = 2928
Division of Time	(1,330)、(331,360)	Day 1–330 training set, Days 331–360 test set

**Fig 5:**
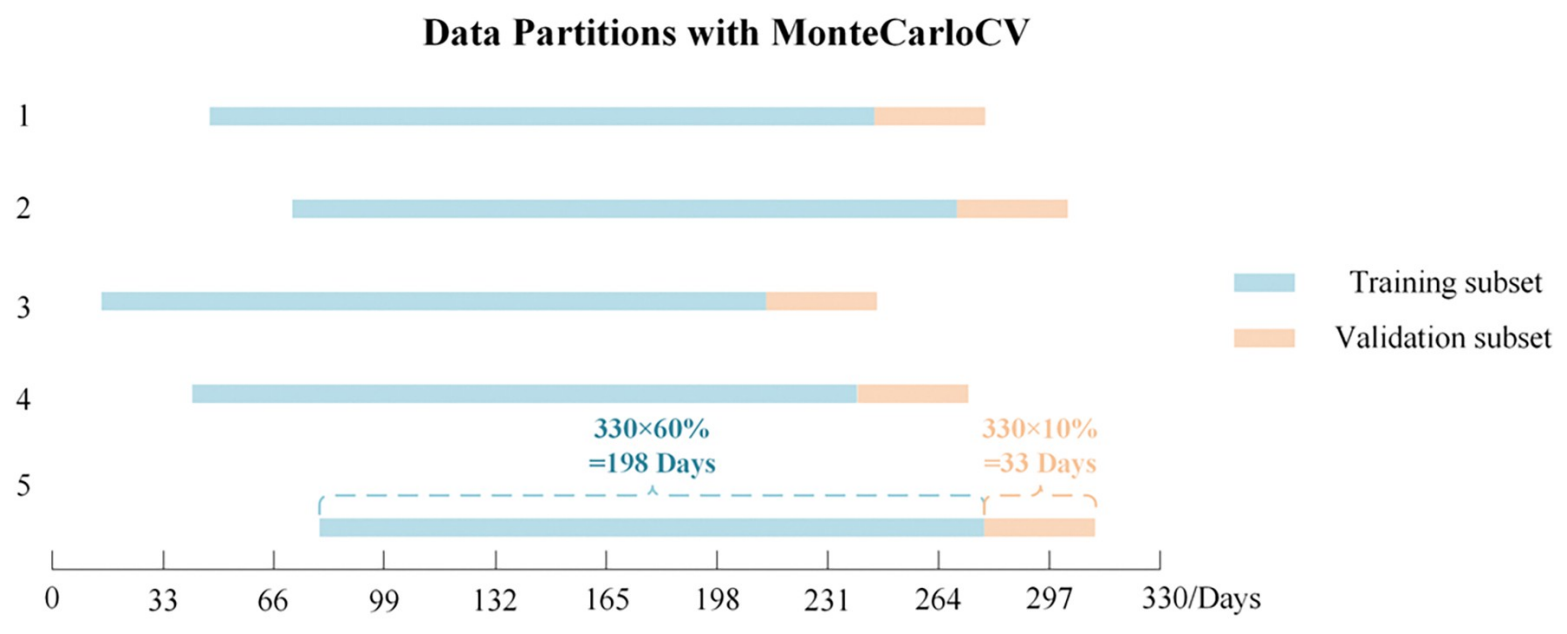
Monte Carlo Time Series Cross-Validation data sets during the first 330 days.

For experiments, the first 330 days of data were used for model training, and the remaining days were divided into a 30-day testing period following the 330-day training period. Due to the limited amount of data available in the training set, over-fitting during model training was a concern. Therefore, Monte Carlo Time Series Cross-Validation (MCTS-CV) was applied to the training data, as illustrated in Fig 5. The approach involves two steps. First, 60% of the time series data from the 330 days were randomly selected as the training set, and 10% were selected as the validation set. No temporal gaps existed between the training and validation sets, and the sample size for the two sets remained constant and contiguous. Second, five iterations were set, in which the starting point for the testing set was randomly selected five times to yield five different sets of training and validation data. The model was trained on each set, which constituted different time-series data, for model evaluation.

### b. Canonical correlation analysis

Multiple air pollutant concentration data from different cities were analyzed for correlation, revealing significant correlation between various air pollutants in different cities. Air pollutant concentration data exhibit temporal correlation and correlation not only between different types of pollutants (PM2.5, PM10, NO2, SO2, CO, and O3) but also between different cities, constituting a spatio-temporal sequence of data with multiple factors affecting the correlation. Therefore, this step employs Canonical Correlation Analysis (CCA), a typical method for analyzing and quantifying the correlation between two sets of variables, to measure the strength of the correlation between the predicted values of multiple air pollutants in various cities and the historical values of multiple pollutants in different cities.

*t* values at the current time of *M* kinds of air pollutants in *N* cities and *p* values at historical times were selected as *X* group data set. The number of variables in *X* group is N*M*(*p*+1). *t*+1 time values of *M* kinds of air pollutants in *N* cities are selected as group *Y*, and the number of variables in group*Y* is N*M. The two groups of variables are shown in Formulas (25) and (26):

$$X=\left({Var}_{11}(t),\cdots ,{{Var}_{NM}(t),\cdots ,Var}_{ij}(t-1),\cdots ,{Var}_{11}(t-p){,\cdots ,Var}_{NM}(t-p)\right)$$ (25)

$$Y=({Var}_{11}(t+1),\cdots ,{Var}_{ij}(t+1),\cdots ,{Var}_{NM}(t+1))$$ (26)

Where, *t*, *t*−1,⋯,*t*−*p* represents the current moment *t* and *p* historical moments, *t*+1 represents the next moment, *i* = 1,2,⋯,*N*, *j* = 1,2,⋯,*M*, *Var* represents the concentration of atmospheric pollutants, *Var*_*ij*_(*t*−1) represents the concentration of the *j*th air pollutant in the *i*th city at time *t*−1.

The first pair of typical variables *x*_1_ and *y*_1_ can be obtained by linear combination of the two groups of variables *X* and *Y*, as shown in Formula (27):

$$\left\{ \begin{array}{c} x_{1}={a0}_{11}{Var}_{11}(t)+\cdots +{a1}_{ij}{Var}_{ij}(t-1)+\cdots +{ap}_{NM}{Var}_{NM}(t-p)\\ y_{1}=b_{11}{Var}_{11}(t+1)+\cdots +b_{ij}{Var}_{ij}(t+1)+\cdots +b_{NM}{Var}_{NM}(t+1) \end{array}\right.$$ (27)

Where {*a*0_11_,⋯,*a*1_*ij*_,⋯*ap*_*NM*_} represents the typical coefficient of group *X*, {*b*_11_,⋯,*b*_*ij*_⋯,*b*_*NM*_} represents the typical coefficient of group *Y*.

Using Pearson’s correlation coefficient calculation formula, the typical correlation coefficients *P*_1_ of the first pair of typical variables *x*_1_ and *y*_1_ are obtained as follows:

$$P_{1}=P(x_{1},y_{1})=\frac{Cov(x_{1},y_{1})}{\sqrt{Var(x_{1})\times Var(y_{1})}}$$ (28)

Where, *Cov* is to obtain covariance and *Var* is to obtain standard deviation.

Formula (27) and (28) are repeated until step *r* is reached, and the correlation between *X* and *Y* variables is extracted, where *r* = *min*(number of variables in *X*, number of variables in *Y*). The typical variable of *r* pair is obtained, and the typical load coefficient and cross load coefficient of *r* pair typical variable and two groups of variables *X* and *Y* are analyzed. The typical-load coefficient represents the simple correlation coefficient of typical variable *x*_*r*_ and *X* group variable, and typical variable *y*_*r*_ and *Y* group variable. The cross load coefficient represents the simple correlation coefficient between the typical variables *x*_*r*_ and *Y*, and between the typical variables *y*_*r*_ and *X*. The larger the absolute value of the load coefficient is, the stronger the correlation between this item and the typical variable is.

When you do correlation analysis, you can look at the typical load coefficient and you can look at the cross load coefficient. For example, among the N*M*(*p*+1) typical load coefficients generated by *x*_1_ and *X*, the absolute values of the first and *N*th coefficients are the highest; Among N*M typical load coefficients generated by *y*_1_ and *Y*, the absolute values of the second and *M*th coefficients are the highest. So that the first and *N*th variables in the *X* group have a strong correlation with the second and *M*th variables in the *Y* group.

Validate using the data set of Table 1 for the Yangtze River Delta City Cluster., *N* = 7、*M* = 6、*p* = 8, the number of variables in group *X* is 7×6×8 = 336, and the number of variables in group *Y* is 7×6 = 42. 42 pairs of typical variables are constituted to determine whether there is correlation between the concentration of 6 kinds of air pollutants in 7 cities at the moment to be predicted and the concentration values of 6 kinds of air pollutants in 7 cities at 8 historical moments, that is, the concentration data of multiple air pollutants in multiple cities have temporal and spatial correlation. Table 3 shows the typical correlation coefficient calculated by the test data.

**Table 3:** Typical correlation analysis of test data.

Canonical variable pair	Canonical correlation coefficient	Canonical variable pair	Canonical correlation coefficient	Canonical variable pair	Canonical correlation coefficient
1st	0.973	15th	0.809	29th	0.674
2nd	0.963	16th	0.798	30th	0.659
3rd	0.932	17th	0.796	31th	0.654
4th	0.927	18th	0.786	32th	0.647
5th	0.905	19th	0.762	33th	0.634
6th	0.9	20th	0.761	34th	0.623
7th	0.889	21th	0.756	35th	0.608
8th	0.88	22th	0.731	36th	0.601
9th	0.871	23th	0.715	37th	0.587
10th	0.854	24th	0.709	38th	0.57
11th	0.847	25th	0.705	39th	0.563
12th	0.833	26th	0.701	40th	0.538
13th	0.829	27th	0.688	41th	0.508
14th	0.813	28th	0.676	42th	0.48

**Fig 6:**
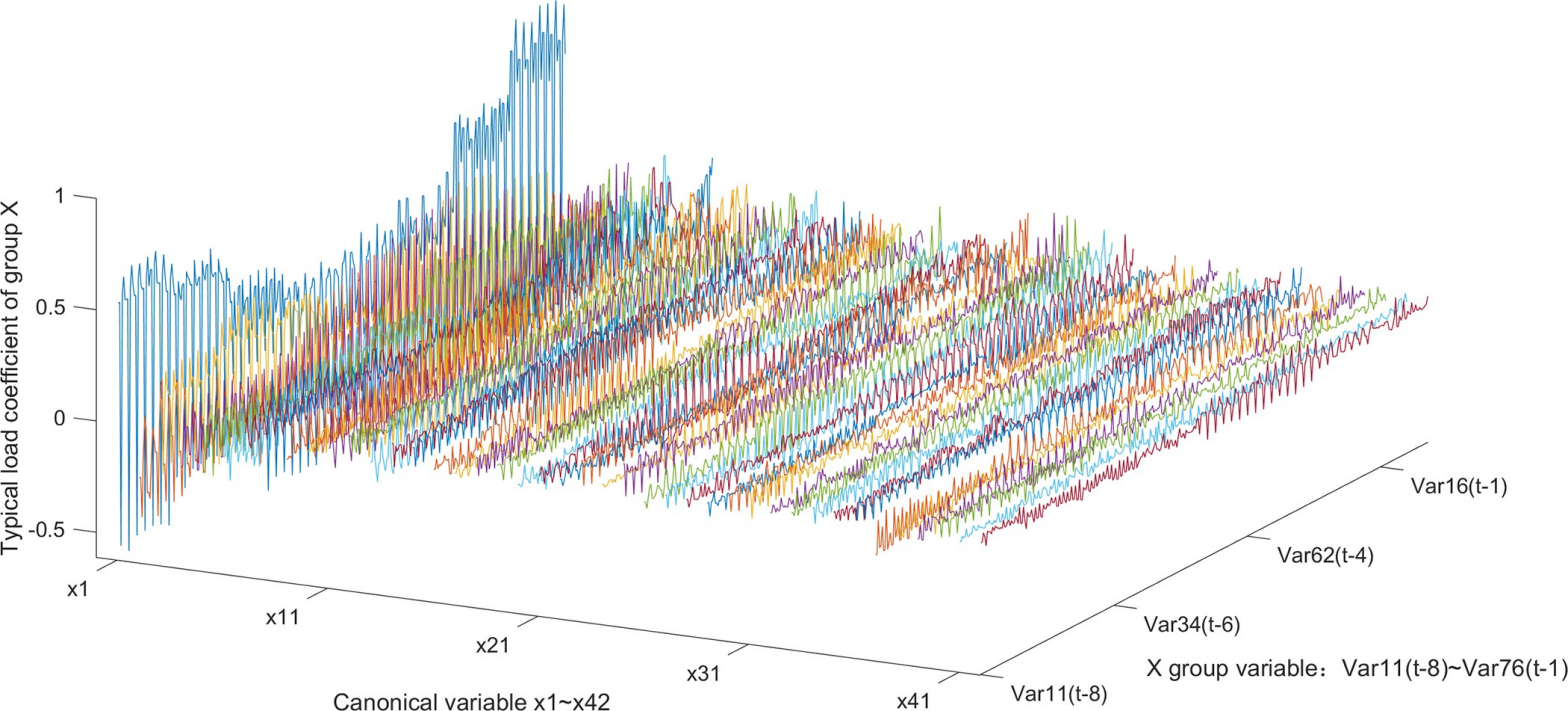
Main view of 3D visual curve of the typical-load coefficient of *x*_*r*_ and *X*.

**Fig 7:**
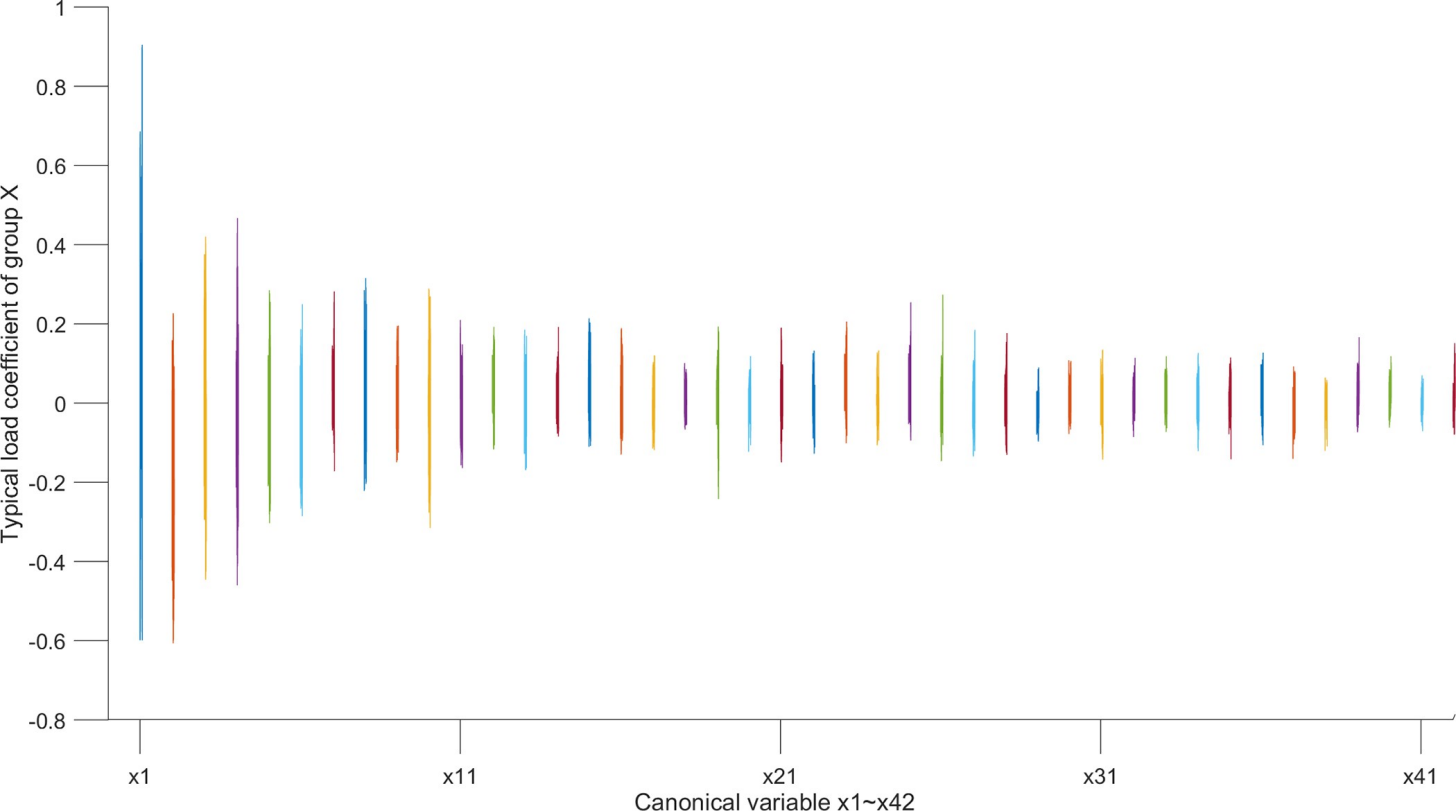
Left view of 3D visual curve of the typical-load coefficient of *x*_*r*_ and *X*.

**Fig 8:**
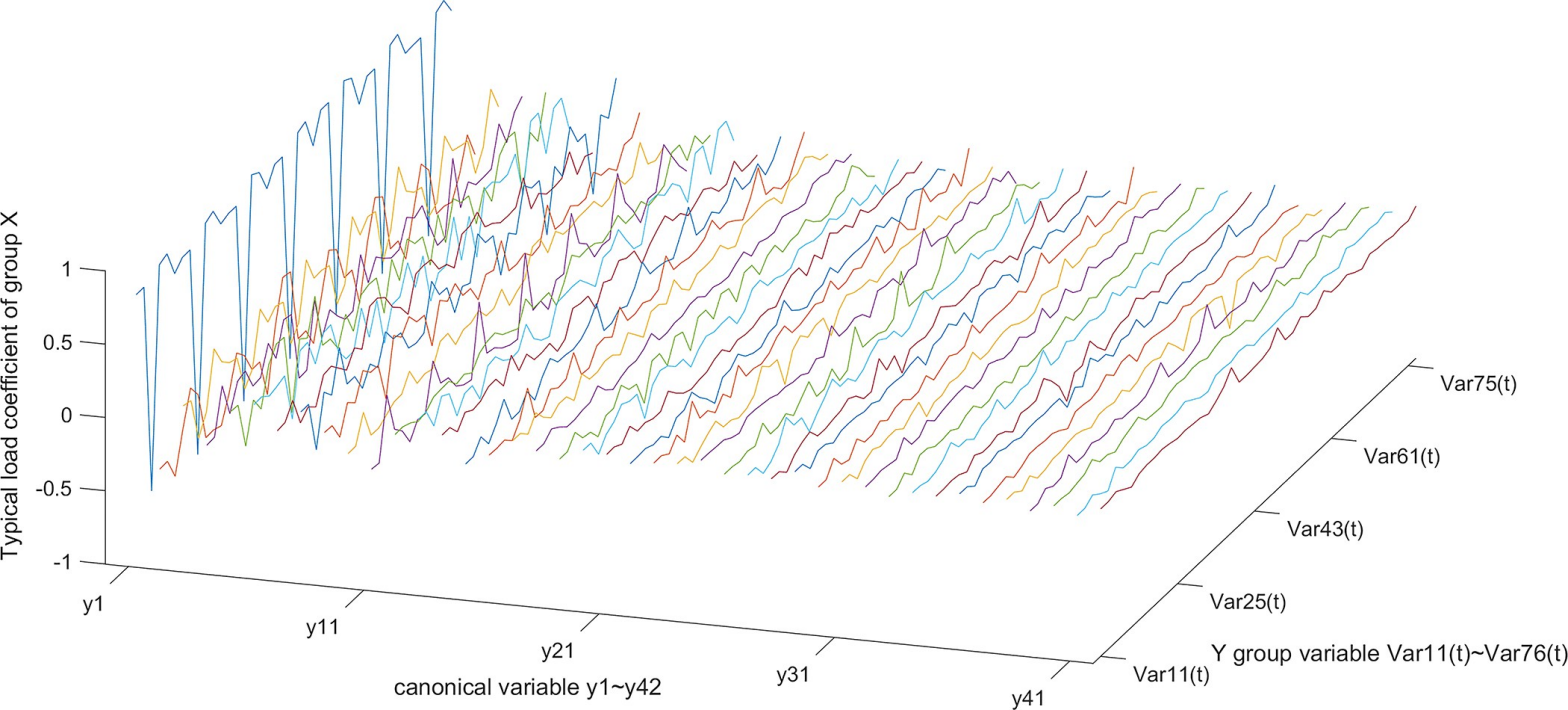
Main view of 3D visual curve of the typical-load coefficient of *y*_*r*_ and *Y*.

**Fig 9:**
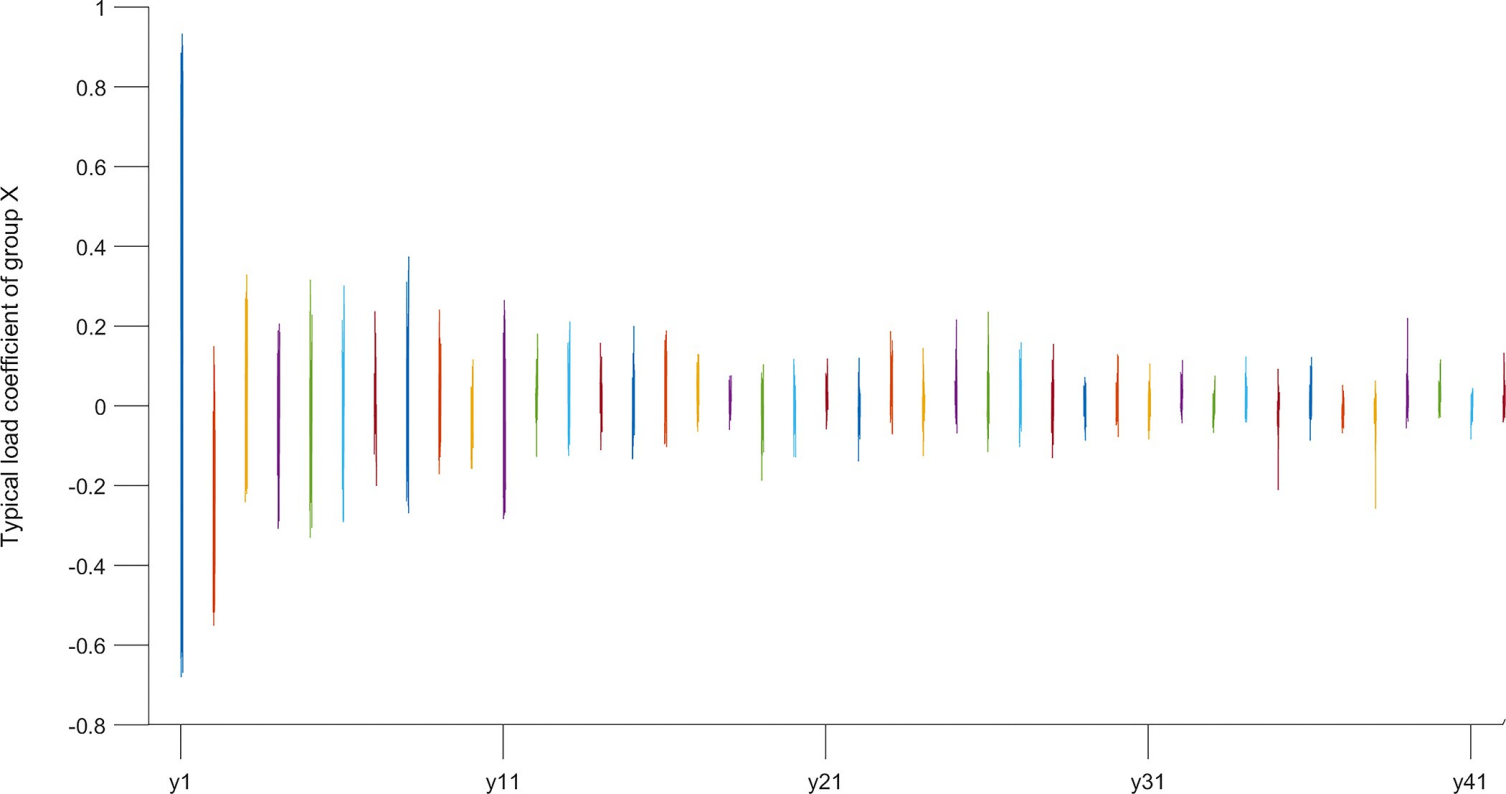
. Left view of 3D visual curve of the typical-load coefficient of *y*_*r*_ and *Y*.

**Fig 10:**
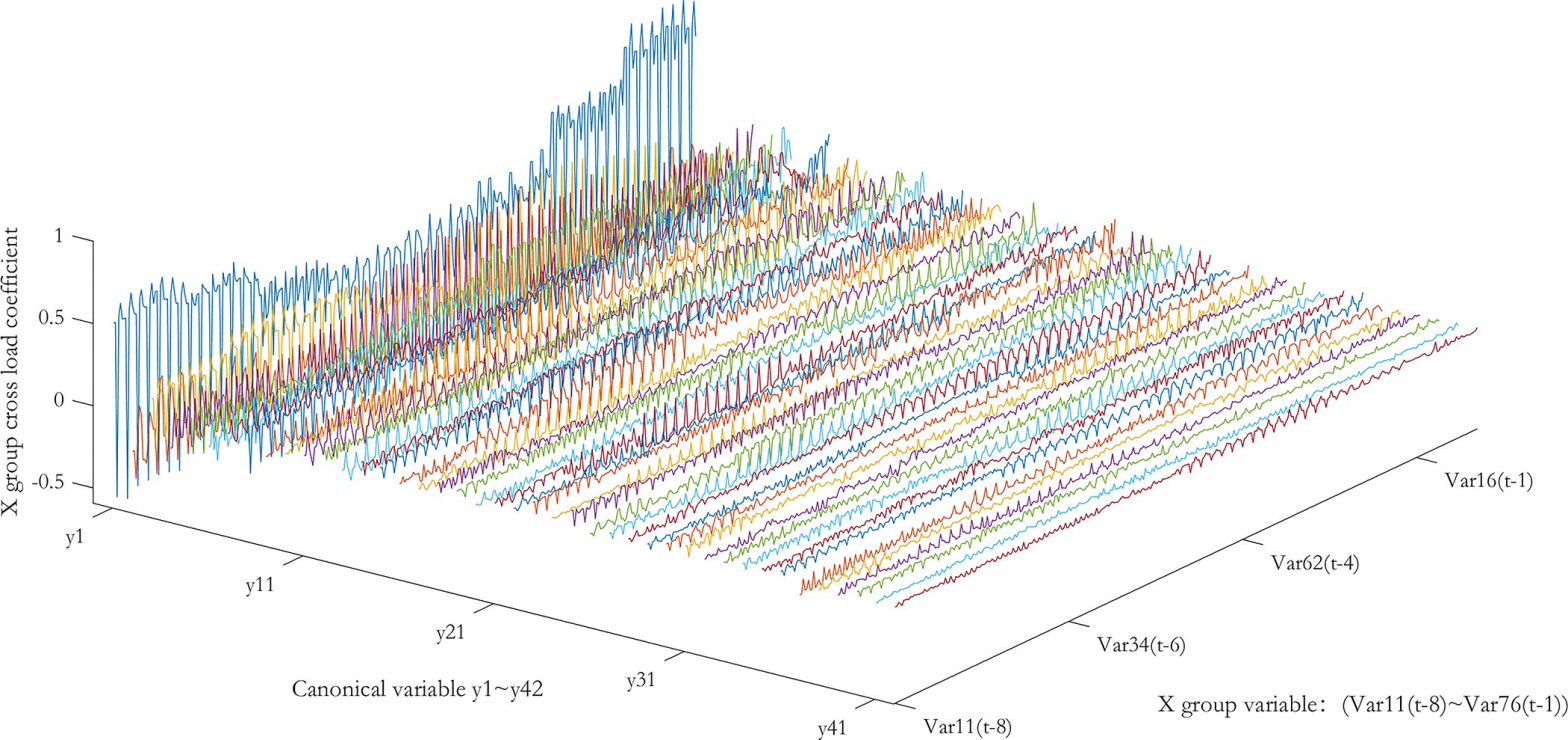
Main view of 3D visual curve of the cross-load coefficient of *y*_*r*_ and *X*.

**Fig 11:**
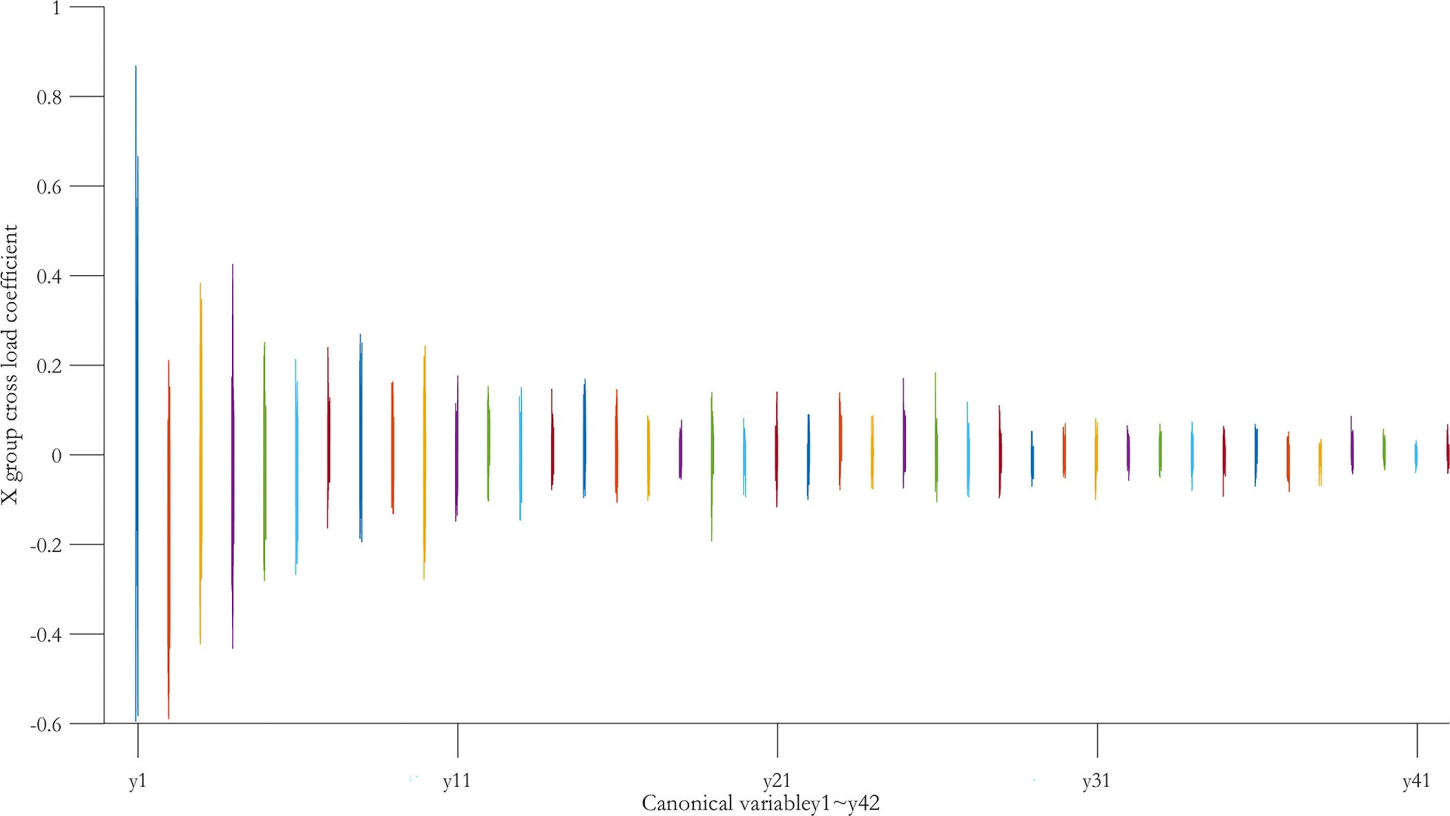
Left view of the 3D visual curve of the cross-load coefficient of *y*_*r*_ and *X*.

**Fig 12:**
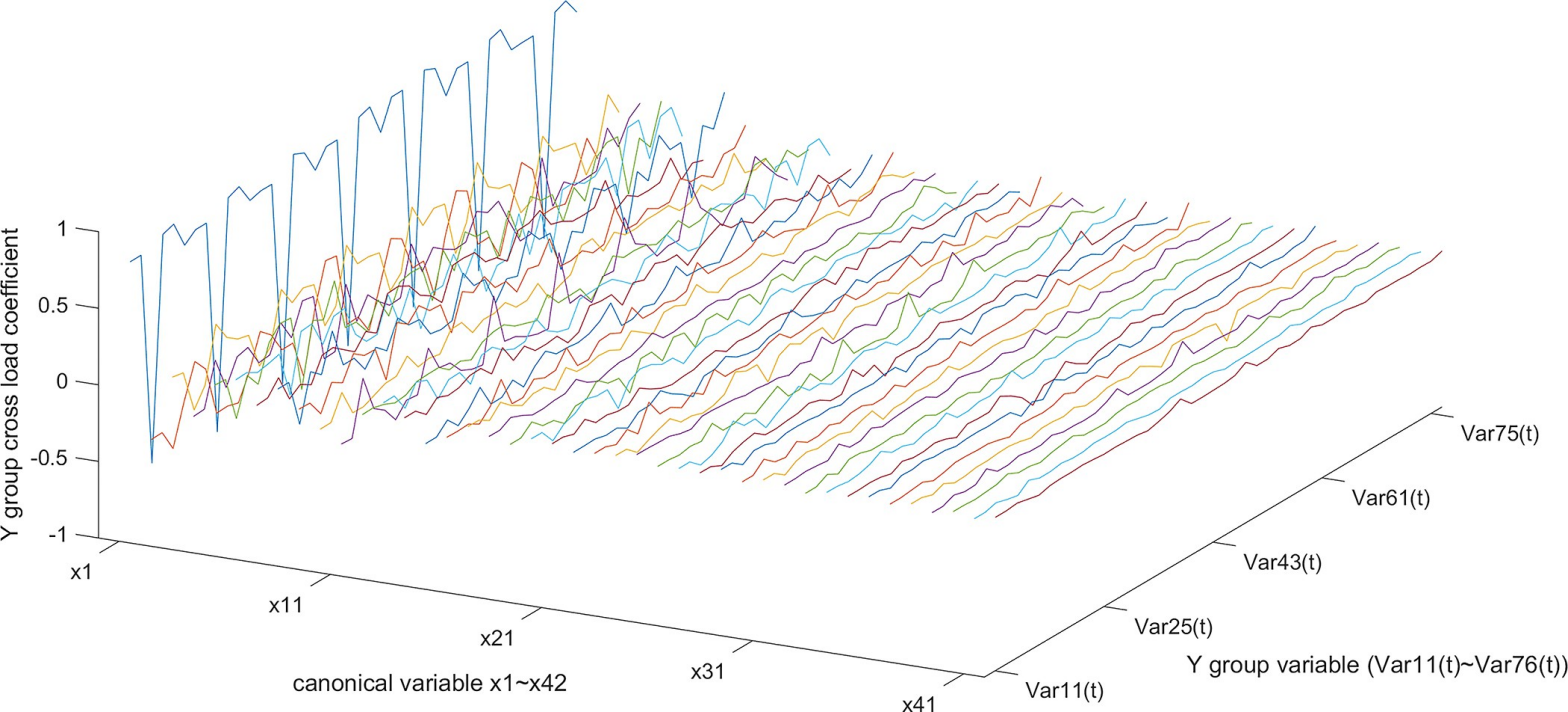
Main view of 3D visual curve of the cross-load coefficient of *x*_*r*_ and *Y*.

**Fig 13:**
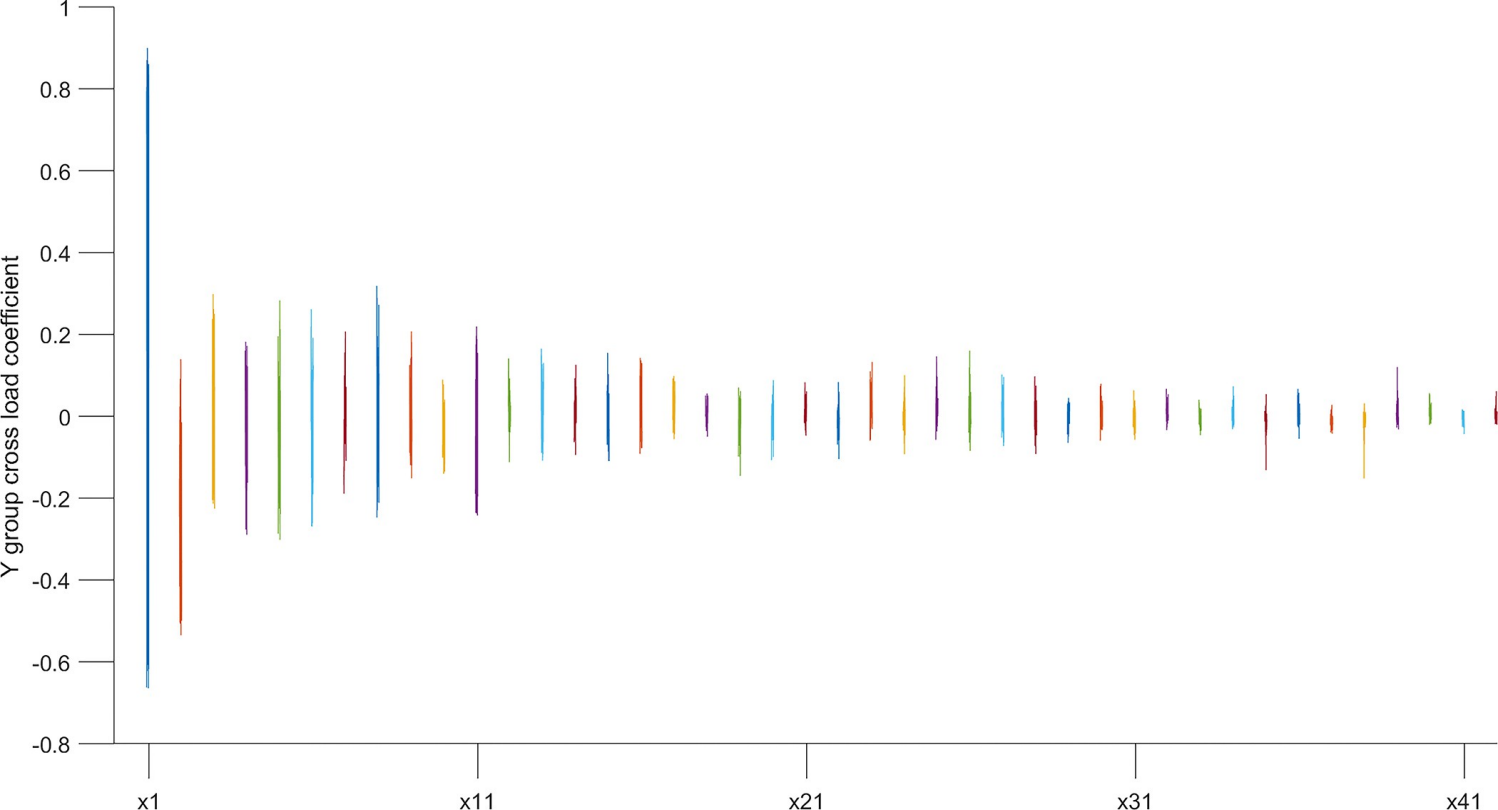
Left view of the 3D visual curve of the cross-load coefficient of *x*_*r*_ and *Y*.

According to the typical load coefficient, the typical result analysis is carried out, and the typical load coefficient is obtained to obtain the correlation between the typical variable and all the variables in this group, that is, the simple correlation coefficient of the typical variable *x*_*r*_ and the *X* group variable as shown in Figs 6 and 7, the simple correlation coefficient of the typical variable *y*_*r*_ and the *Y* group variable as shown in Figs 8 and 9, the cross load coefficient is obtained to obtain the correlation between the typical variable and all the variables in another group, that is, the simple correlation coefficient of the typical variable *x*_*r*_ and the *Y* group variable as shown in Figs 10 and 11, the simple correlation coefficient of the typical variable *y*_*r*_ and the *X* group variable as shown in Figs 12 and 13, and drawn into three-dimensional curves.

As can be seen from the analysis results of the above typical correlation analysis method, the first pair of typical variables *x*_1_ and *y*_1_ and the typical load coefficient and cross load coefficient of the two groups of variables *X* and *Y* are greater than 0.6 respectively, indicating a high correlation between the spatial and temporal correlation of the concentration data of various air pollutants in different cities.

### c. Forecasting performance evaluation and parameters of network

MAE, MAPE and RMSE were used to predict the model results. Define the predicted value as: $\hat{z}=\{{\hat{z}}_{1},{\hat{z}}_{2},\cdots ,{\hat{z}}_{n}\}$, and the true value as: ***z*** = {*z*_1_, *z*_2_,⋯,*z*_*n*_}.

MAE (Mean Absolute Error) calculation formula is as follows:

$$MAE=\frac{1}{n}\sum_{i=1}^{n}\left|{\hat{z}}_{i}-z_{i}\right|$$ (29)

MAPE (Mean Absolute Percentage Error) calculation formula is as follows:

$$MAPE=\frac{1}{n}\sum_{i=1}^{n}|\frac{{\hat{z}}_{i}-z_{i}}{z_{i}}|\times 100\%$$ (30)

RMSE (Root Mean Square Error) calculation formula is as follows:

$$RMSE=\sqrt{\frac{1}{n}\sum_{i=1}^{n}{\left({\hat{z}}_{i}-z_{i}\right)}^{2}}$$ (31)

The learning rate of the model is set to 0.1, the regularization parameter is set to 0, the Dropout parameter is set to 0.5, and the activation function is set to ReLU. The Epoch is set to 250 times, and the Batch is set to 64.

## 4. Results and discussions

### a. Concrete structure of four-dimensional directed GCN

Firstly, a two-dimensional plane coordinate axis is established according to the longitude and latitude information of seven cities, as shown in Fig 3.

Each city is the node of ***G***: ***ν*** = {***v***_**1**_,⋯,***v***_**7**_}, edge ***e*** of ***G*** is represented by an internode vector. City ***v***_**1**_ defines the city ***v***_**2**_ spatial association as edge $e_{12}:\vec{v_{1}v_{2}}=(x_{v2}-x_{v1},y_{v2}-y_{v1})=(32.01-31.30,120.86-120.63)=(0.71,0.23)$, City ***v***_**2**_ defines the city ***v***_**1**_ spatial association as edge $e_{21}:\vec{v_{2}v_{1}}=(x_{v1}-x_{v2},y_{v1}-y_{v2})=(31.30-32.01,120.63-120.86)=(-0.71,-0.23)$, by the same token, the adjacency ***A*** of ***G*** is:

$$\left(\begin{array}{ccccccc} \left(0,0\right) & \left(0.71,0.23\right) & \left(0.71,0.23\right) & \left(0.71,0.23\right) & \left(-0.52,0.21\right) & \left(-0.55,0.16\right) & \left(-0.45,0.86\right)\\ \left(0.71,0.23\right) & \left(0,0\right) & \left(-0.29,-0.76\right) & \left(-0.67,-1.04\right) & \left(-1.23,-0.44\right) & \left(-1.26,-0.07\right) & \left(-1.16,0.63\right)\\ \left(0.71,0.23\right) & \left(0.29,0.76\right) & \left(0,0\right) & \left(-0.38,-0.28\right) & \left(-.94,0.32\right) & \left(-0.97,0.69\right) & \left(-0.87,1.39\right)\\ \left(0.71,0.23\right) & \left(0.67,1.04\right) & \left(-0.38,-0.28\right) & \left(0,0\right) & \left(-0.56,0.6\right) & \left(-0.59,0.97\right) & \left(-0.49,1.67\right)\\ \left(-0.52,0.21\right) & \left(1.23,0.44\right) & \left(-.94,0.32\right) & \left(0.56,-0.6\right) & \left(0,0\right) & \left(0.03,0.37\right) & \left(0.07,1.07\right)\\ \left(-0.55,0.16\right) & \left(1.26,0.07\right) & \left(-0.97,0.69\right) & \left(0.59,-0.97\right) & \left(0.03,0.37\right) & \left(0,0\right) & \left(0.1,0.7\right)\\ \left(-0.45,0.86\right) & \left(1.16,-0.63\right) & \left(-0.87,1.39\right) & \left(0.49,-1.67\right) & \left(0.07,1.07\right) & \left(-0.1,-0.7\right) & \left(0,0\right) \end{array}\right)$$

The corresponding degree matrix ***D*** is as follows:

$$\left(\begin{array}{ccccccc} \left(2.69,2.8\right) & \left(0,0\right) & \left(0,0\right) & \left(0,0\right) & \left(0,0\right) & \left(0,0\right) & \left(0,0\right)\\ \left(0,0\right) & \left(5.32,3.17\right) & \left(0,0\right) & \left(0,0\right) & \left(0,0\right) & \left(0,0\right) & \left(0,0\right)\\ \left(0,0\right) & \left(0,0\right) & \left(3.87,3.97\right) & \left(0,0\right) & \left(0,0\right) & \left(0,0\right) & \left(0,0\right)\\ \left(0,0\right) & \left(0,0\right) & \left(0,0\right) & \left(2.73,5.37\right) & \left(0,0\right) & \left(0,0\right) & \left(0,0\right)\\ \left(0,0\right) & \left(0,0\right) & \left(0,0\right) & \left(0,0\right) & \left(3.35,3.01\right) & \left(0,0\right) & \left(0,0\right)\\ \left(0,0\right) & \left(0,0\right) & \left(0,0\right) & \left(0,0\right) & \left(0,0\right) & \left(3.96,2.96\right) & \left(0,0\right)\\ \left(0,0\right) & \left(0,0\right) & \left(0,0\right) & \left(0,0\right) & \left(0,0\right) & \left(0,0\right) & \left(3.14,6.32\right) \end{array}\right)$$

According to the definition of the Laplace matrix, the Laplace matrix ***L*** can be obtained as follows:

$$\left(\begin{array}{ccccccc} \left(2.69,2.8\right) & \left(-0.71,-0.23\right) & \left(-0.42,0.53\right) & \left(-0.04,0.81\right) & \left(0.52,0.21\right) & \left(0.55,-0.16\right) & \left(0.45,-0.86\right)\\ \left(0.71,0.23\right) & \left(5.32,3.17\right) & \left(0.29,0.76\right) & \left(0.67,1.04\right) & \left(1.23,0.44\right) & \left(1.26,0.07\right) & \left(1.16,-0.63\right)\\ \left(0.42,0.53\right) & \left(-0.29,-0.76\right) & \left(3.87,3.97\right) & \left(0.38,0.28\right) & \left(0.94,-0.32\right) & \left(0.97,-0.69\right) & \left(0.87,-1.39\right)\\ \left(0.04,0.81\right) & \left(-0.67,-1.04\right) & \left(-0.38,0.32\right) & \left(2.73,5.37\right) & \left(0.56,-0.6\right) & \left(0.59,-0.97\right) & \left(0.49,-1.67\right)\\ \left(-0.52,0.21\right) & \left(-1.23,-0.44\right) & \left(-0.94,0.69\right) & \left(-0.56,0.6\right) & \left(3.35,3.01\right) & \left(-0.03,-0.37\right) & \left(-0.07,-1.07\right)\\ \left(-0.55,0.16\right) & \left(-1.26,-0.07\right) & \left(-0.97,1.39\right) & \left(-0.59,0.97\right) & \left(-0.49,1.67\right) & \left(3.96,2.96\right) & \left(-0.1,-0.7\right)\\ \left(-0.45,0.86\right) & \left(-1.16,0.63\right) & \left(-0.87,1.39\right) & \left(-0.49,1.67\right) & \left(0.07,1.07\right) & \left(0.1,0.7\right) & \left(3.14,6.32\right) \end{array}\right)$$

In the calculation of graph convolutional in the spectral domain, spectral decomposition of the Laplacian matrix of the graph is needed to obtain the basis of the Fourier transform of the graph. On this premise, it is necessary to ensure that the Laplacian matrix of the graph is a symmetric matrix before spectral decomposition of the matrix. Therefore, by using the properties of symmetric matrix and antisymmetric matrix 3, namely Formula (3), A new form of graph adjacency matrix ***A***′ is obtained by transforming the original directed graph into a new undirected graph by formula transformation and preserving the spatial direction information of nodes in the directed graph. The adjacency matrix ***A***′ after formula transformation is as follows:

$$\left(\begin{array}{ccccccc} \left(0,0\right) & \left(-1.8673,-0.0851\right) & \left(-0.4956,0.6201\right) & \left(-0.0016.2.0817\right) & \left(0.3432,0.0441)\right) & \left(0.4455,-0.0256\right) & \left(0.2025,-3.0272\right)\\ \left(-1.8673,-0.0851\right) & \left(0.0\right) & \left(-0.4205,0.6080\right) & \left(-1.7353,2.2880\right) & \left(-2.4231.-0.0704\right) & \left(-2.2932,-0.0147\right) & \left(-0.6351,-1.9845\right)\\ \left(-0.4956,0.6201\right) & \left(-0.4205,0.6080\right) & \left(0,0\right) & \left(-0.4332,0.3920\right) & \left(-0.4888,0.3072\right) & \left(-0.3589,0.6969\right) & \left(-0.6351,-3.2665\right)\\ \left(-0.0016,2.0817\right) & \left(-1.7353,2.2880\right) & \left(-0.4332,0.3920\right) & \left(0,0\right) & \left(0.3472,1.4160\right) & \left(0.4543,2.3377\right) & \left(0.2009,-1.5865\right)\\ \left(0.3432,0.0441\right) & \left(-2.4231,-0.0704\right) & \left(-0.4888,0.3072\right) & \left(0.3472.1.4160\right) & \left(0,0\right) & \left(0.0045,0.0185\right) & \left(0.0147,-3.5417\right)\\ \left(0.4455,-0.0256\right) & \left(-2.2932,-0.0147\right) & \left(-0.3589,0.6969\right) & \left(0.4543,2.3377\right) & \left(0.0045,0.0185\right) & \left(0.0\right) & \left(0.0360,-2.3520\right)\\ \left(0.2025,-3.0272\right) & \left(-0.6351,-1.9845\right) & \left(-0.6351,-3.2665\right) & \left(0.2009,-1.5865\right) & \left(0.0147,-3.5417\right) & \left(0.0360,-2.3520\right) & \left(0,0\right) \end{array}\right)$$

The new adjacency matrix is obtained as a symmetric matrix by transformation, and its degree matrix ***D***′ is obtained by corresponding update, as follows:

$$\left(\begin{array}{ccccccc} \left(3.3557,5.8838\right) & \left(0,0\right) & \left(0,0\right) & \left(0,0\right) & \left(0,0\right) & \left(0,0\right) & \left(0,0\right)\\ \left(0,0\right) & \left(11.2682,5.0507\right) & \left(0,0\right) & \left(0,0\right) & \left(0,0\right) & \left(0,0\right) & \left(0,0\right)\\ \left(0.0\right) & \left(0,0\right) & \left(2.8321,5.8907\right) & \left(0,0\right) & \left(0,0\right) & \left(0,0\right) & \left(0.0\right)\\ \left(0,0\right) & \left(0,0\right) & \left(0,0\right) & \left(3.1725,10.1019\right) & \left(0,0\right) & \left(0,0\right) & \left(0,0\right)\\ \left(0,0\right) & \left(0,0\right) & \left(0,0\right) & \left(0,0\right) & \left(3.6215,5.3979\right) & \left(0,0\right) & \left(0,0\right)\\ \left(0,0\right) & \left(0,0\right) & \left(0,0\right) & \left(0,0\right) & \left(0,0\right) & \left(3.5924,5.4454\right) & \left(0,0\right)\\ \left(0.0\right) & \left(0,0\right) & \left(0,0\right) & \left(0,0\right) & \left(0,0\right) & \left(0,0\right) & \left(3.6180,15.7584\right) \end{array}\right)$$

Then, according to Formula (4), the Laplace matrix ***L***′ can be obtained, and the corresponding eigenvalues ***Λ*** and eigenvectors ***U*** can be calculated by spectral decomposition.

Set the parameter ***k*** = **3** of the graph filter according to Formula (9), and the following formula can be obtained:

$${Her}_{3}(\Lambda )=2\Lambda ∙{Her}_{2}(\Lambda )-4{Her}_{1}(\Lambda )$$

$$=2\Lambda ∙(2\Lambda ∙{Her}_{1}(\Lambda )-2{Her}_{0}(\Lambda ))-4{Her}_{1}(\Lambda )$$

$$=8\Lambda^{3}-12\Lambda$$ (32)

Then, by substituting Formula (32) into Formula (10) and setting parameter ***θ***_***k***_ as 1, the following formula can be obtained:

$$g_{\theta }^{\prime }(\Lambda )=\sum_{\kappa =0}^{3}\theta_{3}{Her}_{3}(\Lambda )$$

$$=\theta_{0}{Her}_{0}(\Lambda )+\theta_{1}{Her}_{1}(\Lambda )+\theta_{2}{Her}_{2}(\Lambda )+\theta_{3}{Her}_{3}(\Lambda )$$

$$=E+2\Lambda +2(2\Lambda^{2}-\Lambda )+4(2\Lambda^{3}-3\Lambda )$$

$$=8\Lambda^{3}+4\Lambda^{2}-12\Lambda +E$$ (33)

Where ***E*** is the identity matrix. Finally, Formula (33) is substituted into Formula (11) to obtain the input signal:

$$H*g_{\theta }^{\prime }(\Lambda )$$

$$=U(\theta_{0}M_{0}(\Lambda )+\theta_{1}M_{1}(\Lambda )+\theta_{2}M_{2}(\Lambda )+\theta_{3}M_{3}(\Lambda ))U^{Τ}∙H$$

$$=U(E+2\Lambda +2(2\Lambda^{2}-\Lambda )+4(2\Lambda^{3}-3\Lambda ))U^{Τ}∙H$$

$$=U(8\Lambda^{3}+4\Lambda^{2}-12\Lambda +E)U^{Τ}∙H$$

### b. Prediction results of 4D-DGCN-LSTM

The constructed 4D-DGCN-LSTM model is used to predict air pollution. The working process of predicting the concentration of various air pollutants in multiple cities is as follows:

The constructed 4D-DGCN-LSTM model is used to predict the concentration of atmospheric pollutants at time ***t***+**1**. Determine the input of the model for {***X***_***t*−*p***_,⋯,***X***_***t***−**1**_,***X***_***t***_} is composed of the current time ***t*** and ***p*** historical time concentration values of ***M*** kinds of air pollutants in ***N*** cities, which are specifically expressed as:

$$X_{t-p}=\left(\begin{array}{ccc} {Var}_{11}(t-p) & \cdots & {Var}_{1M}(t-p)\\ \vdots & \ddots & \vdots \\ {Var}_{N1}(t-p) & \cdots & {Var}_{NM}(t-p) \end{array}\right),\cdots ,$$

$$X_{t-1}=\left(\begin{array}{ccc} {Var}_{11}(t-1) & \cdots & {Var}_{1M}(t-1)\\ \vdots & \ddots & \vdots \\ {Var}_{N1}(t-1) & \cdots & {Var}_{NM}(t-1) \end{array}\right),$$

$$X_{t}=\left(\begin{array}{ccc} {Var}_{11}(t) & \cdots & {Var}_{1M}(t)\\ \vdots & \ddots & \vdots \\ {Var}_{N1}(t) & \cdots & {Var}_{NM}(t) \end{array}\right)$$

Where, *Var*_*ij*_(*t*) represents the *j*th atmospheric pollutant concentration in the *i*th city at time *t*, *i* = 1,2,⋯,*N*, *j* = 1,2,⋯,*M*, the four-dimensional directed GCN-LSTM model is used to predict the atmospheric pollutant concentration at time *t*+1. Then the output of the 4D-DGCN-LSTM model at time $t\;{\hat{H}}_{t}$ is the predicted concentration value of *M* kinds of air pollutants in *N* cities at time *t*+1, namely:

$${\hat{H}}_{t}=\left(\begin{array}{ccc} {Var}_{11}(t+1) & \cdots & {Var}_{1M}(t+1)\\ \vdots & \ddots & \vdots \\ {Var}_{N1}(t+1) & \cdots & {Var}_{NM}(t+1) \end{array}\right)$$

In order to demonstrate, the predictive results of PM2.5 concentration in Suzhou and Beijing cities were presented. The comparisons were made in terms of Mean Absolute Error (MAE), Mean Absolute Percentage Error (MAPE) and Root Mean Squared Error (RMSE) metrics for four different models including Long Short-Term Memory (LSTM), Graph Convolutional Network (GCN), Graph Convolutional Network combined with LSTM (GCN-LSTM), and 4D Directed Graph Convolutional Network combined with LSTM (4D-DGCN-LSTM), the evaluation indicators of each model are shown in Tables 4 and 5.

**Table 4:** Evaluation metrics for various models using the data set of Yangtze River Delta City Cluster.

	MAE	MAPE	RMSE
LSTM	25.43	0.44%	38.58
GCN	19.38	0.38%	29.52
GCN-LSTM	18.67	0.37%	28.51
4D-DGCN	14.88	0.31%	25.48
4D-DGCN-LSTM	13.76	0.26%	23.13

**Table 5:** Evaluation metrics for various models using the data set of Yangtze River Delta City Cluster.

	MAE	MAPE	RMSE
LSTM	29.09	0.54%	50.80
GCN	21.11	0.41%	40.02
GCN-LSTM	19.15	0.40%	30.77
4D-DGCN	12.25	0.23%	22.44
4D-DGCN-LSTM	9.36	0.21%	17.02

**Fig 14:**
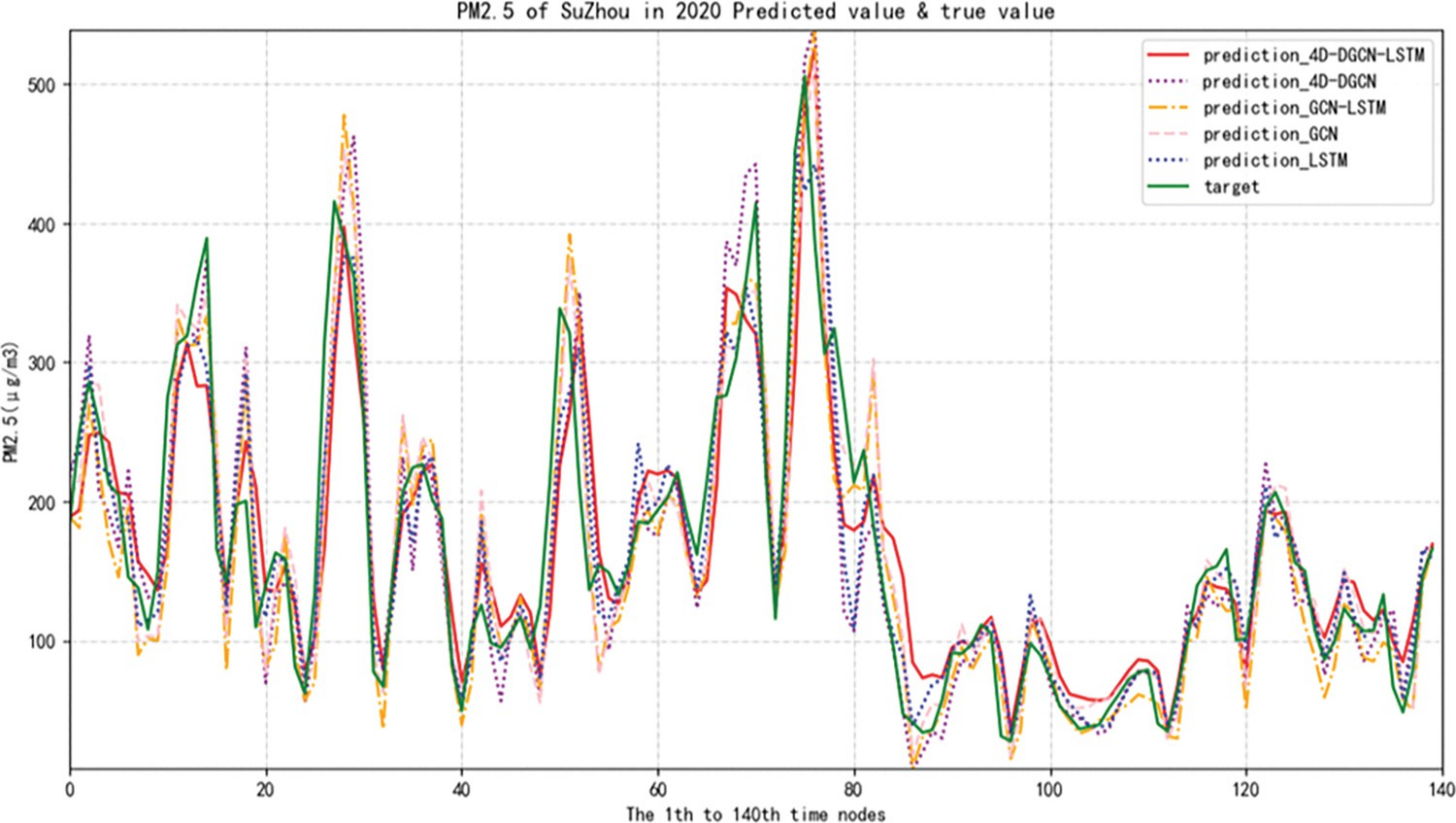
Curve of predicted and true PM2.5 concentration in Suzhou by five methods.

As shown in Fig 14, the red solid line represents the predicted value curve of the proposed method (prediction_4D-DGCN-LSTM), while the green solid line represents the true value curve (target), and the remaining lines represent the predicted value curves of the compared methods.

**Fig 15:**
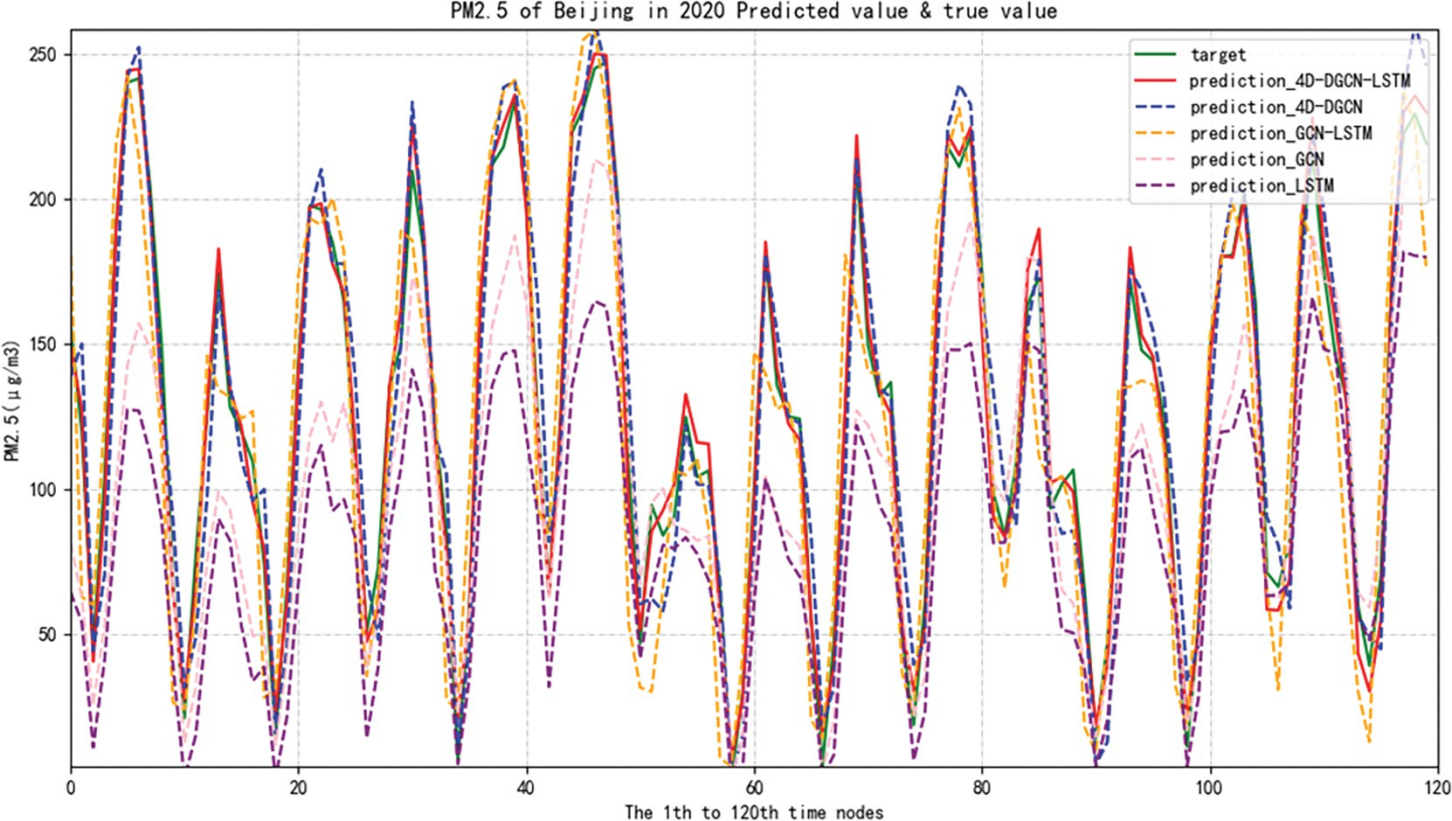
Curve of predicted and true PM2.5 concentration in Beijing by five methods.

As shown in Fig 15, the red solid line represents the predicted value curve of the proposed method (prediction_4D-DGCN-LSTM), while the green solid line represents the true value curve (target), and the remaining lines represent the predicted value curves of the compared methods.

### c. Discussions

The purpose of the experiment is to verify that the proposed 4D-DGCN-LSTM model and the four models 4D-DGCN, GCN-LSTM, GCN, and LSTM are the most effective models for predicting urban air pollutants. (Note: The graph structures of GCN and GCN-LSTM in the compared models use one-dimensional undirected distance graphs.) The following conclusions can be drawn from the analysis of the predicted and true value curves of the PM2.5 concentration in Suzhou in 2020 among the models in Fig 14 and the results of the three error evaluation indicators in Table 4.

Firstly, when comparing the evaluation indicators of the LSTM and GCN models (using MAE as an example), the MAE value of the LSTM model is 25.43%, and the MAE value of the GCN model is 19.38%, which is a decrease of 6.05% compared to the LSTM model. Considering that LSTM is a classic algorithm for handling time-series prediction problems and comparing the prediction results of GCN models for multiple cities and multiple air pollutants, it shows the importance of applying spatial features to the prediction of air pollutants. The method of converting multiple cities and multiple air pollutants into graph structures to extract spatial features is suitable for predicting air pollutants in multiple cities or multiple stations.

Secondly, when comparing the evaluation indicators of GCN and GCN-LSTM models, the MAE value of the GCN model is 19.38%, and the MAE value of the GCN-LSTM model is 18.67%, which is a decrease of 0.71% compared to the GCN model. It shows that it is necessary to embed the long short-term memory network model when using the graph convolutional neural network model to predict the concentration of multiple air pollutants in multiple cities, which can further improve the model’s predictive accuracy.

Thirdly, when comparing the evaluation indicators of GCN and 4D-DGCN models, the MAE value of the GCN model is 19.38%, and the MAE value of the 4D-DGCN model is 14.88%, which is a decrease of 3.41% compared to the GCN model. It shows that if only the distance information between city nodes is used to construct a one-dimensional undirected graph structure, the directional information between cities will be lost when expanding the spatial correlation of multiple cities. However, the 4D-DGCN model uses city longitude and latitude information to construct a two-dimensional directed graph structure, forming a directed graph convolutional neural network model with spatial two-dimensional, time one-dimensional, and multi-factor one-dimensional properties. It not only considers the distance between cities but also the directional angle information between them, greatly improving the model’s predictive accuracy. When predicting substances with spatial diffusivity like air pollutants, it is necessary to extract spatial features and consider the two-dimensionality and directional properties of the graph structure.

Finally, the MAE value of the proposed 4D-DGCN-LSTM model is 13.76%, which is a decrease of 1.12%, 4.91%, 5.62%, and 11.67% compared to the 4D-DGCN, GCN-LSTM, GCN, and LSTM models, respectively. The results show that the 4D-DGCN-LSTM model achieves good results in predicting multiple air pollutants in multiple cities.

## 5. Conclusion

This article focuses on various air pollutants across multiple cities. It analyzes the correlation between air pollutants and factors such as time and space, and proposes a 4D-D GCN-LSTM-based method for predicting air pollutants across multiple cities. The method uses big data to predict concentrations of six air pollutants across cities like Suzhou and Shanghai. Based on the results of this example verification, the article concludes that…

1. For predicting concentrations of various air pollutants in multiple cities, extracting spatial features is crucial. In the past, GCN has been used to address such problems by defining the edges of the graph structure using geographic distance, resulting in a one-dimensional, undirected graph structure. This method lacks accuracy in depicting the spatial relationship between nodes in the topology of urban areas, thus ignoring some spatial information. Therefore, it is necessary to develop a directed graph convolutional neural network that includes two-dimensional structures to better represent the spatial information.
2. To capture the two-dimensional directedness of air pollutants in spatial space, this study employs a two-dimensional directed graph to topologically represent the spatial correlation of air pollutants among multiple cities and proposes a 4D-DGCN model. To address the inability to directly decompose and convolve the spectral domain in a directed graph and tensor calculation, a corresponding two-dimensional directed graph spectral decomposition and tensor calculation algorithm is presented. This algorithm enables spectral domain graph convolutional on the two-dimensional directed graph, providing more spatial correlation information for air pollutant concentration prediction. Based on this, the 4D-DGCN model is embedded in the LSTM model, creating the 4D-DGCN-LSTM model that unifies multiple-factor correlation, time correlation, and spatial correlation of air pollutants for modeling.
